# Larger Real-World OCT Reference Database Improves Accuracy of Glaucoma Flagging Using Summary Metrics

**DOI:** 10.1167/tvst.15.3.6

**Published:** 2026-03-09

**Authors:** Donald C. Hood, Mary Durbin, Chris Lee, Anya Guzman, Tayna Gebhardt, Yujia Wang, Carlos Gustavo De Moraes, Emmanouil Tsamis

**Affiliations:** 1 Bernard and Shirlee Brown Glaucoma Research Laboratory, Department of Ophthalmology, Edward S. Harkness Eye Institute, Columbia University Irving Medical Center, New York, NY, USA; 2 Department of Psychology, Columbia University, New York, NY, USA; 3 Topcon Corporation, Oakland, NJ, USA

**Keywords:** optical coherence tomography, glaucoma, reference database, real-world data

## Abstract

**Purpose:**

To compare the healthy control and glaucoma eyes flagged as outside normal limits (yellow or red) by a commercial reference database (C-RDB) and a larger real-world (RW)–RDB.

**Methods:**

The C-RDB consisted of 398 eyes/individuals. Based only on optical coherence tomography (OCT) reports, the RW-RDB consisted of 4830 eyes/individuals selected from optometry practices using a reading center method. The fifth and first percentile quantile regression lines (QRLs) versus age were calculated for both RDBs for common OCT metrics, including global circumpapillary retinal nerve fiber (g-cpRNFL) and global ganglion cell layer plus inner plexiform layer (g-GCL+) thickness. The test dataset contained 175 healthy control (H) eyes and 183 eyes with OCT defects consistent with optic neuropathy–glaucoma (ON-G). These eyes were flagged as yellow (red) if they fell below the fifth (first) percentile QRL. The QRLs were also compared to a Gaussian model and Monte Carlo simulations.

**Results:**

The C-RDB and RW-RDB did not flag an identical set of eyes as red or yellow. In fact, 16% (g-cpRNFL) and 7% (g-GCL+) of the 183 ON-G eyes had a different color flag. The results of the model and simulations support the hypothesis that both RDBs are sampled from essentially the same underlying “normal” population. Thus, the difference between them is largely due to less random error in the larger sample.

**Conclusions:**

There is a difference in the eyes flagged, and this difference is largely due to the greater size of the RW-RDB.

**Translational Relevance:**

These findings support the clinical value of expanding reference databases to improve diagnostic accuracy of glaucoma flagging.

## Introduction

Optical coherence tomography (OCT) is becoming commonplace in the diagnosis and monitoring of glaucoma. Since its inception, OCT glaucoma reports have featured summary statistics, which we will refer to as metrics. In OCT and glaucoma, the most common metrics include global (average) circumpapillary retinal nerve fiber layer (g-cpRNFL) thickness and global (average) ganglion cell plus inner plexiform layer (g-GCL+) thickness of the central ±10°. These metrics are widely used by clinicians to aid in decision-making regarding the presence or progression of glaucoma. Additionally, clinical trials that incorporate OCT commonly rely on these metrics, and variations of them have been proposed as essential components of glaucoma definitions in research studies.^1^^–^^3^

**Figure 1.**
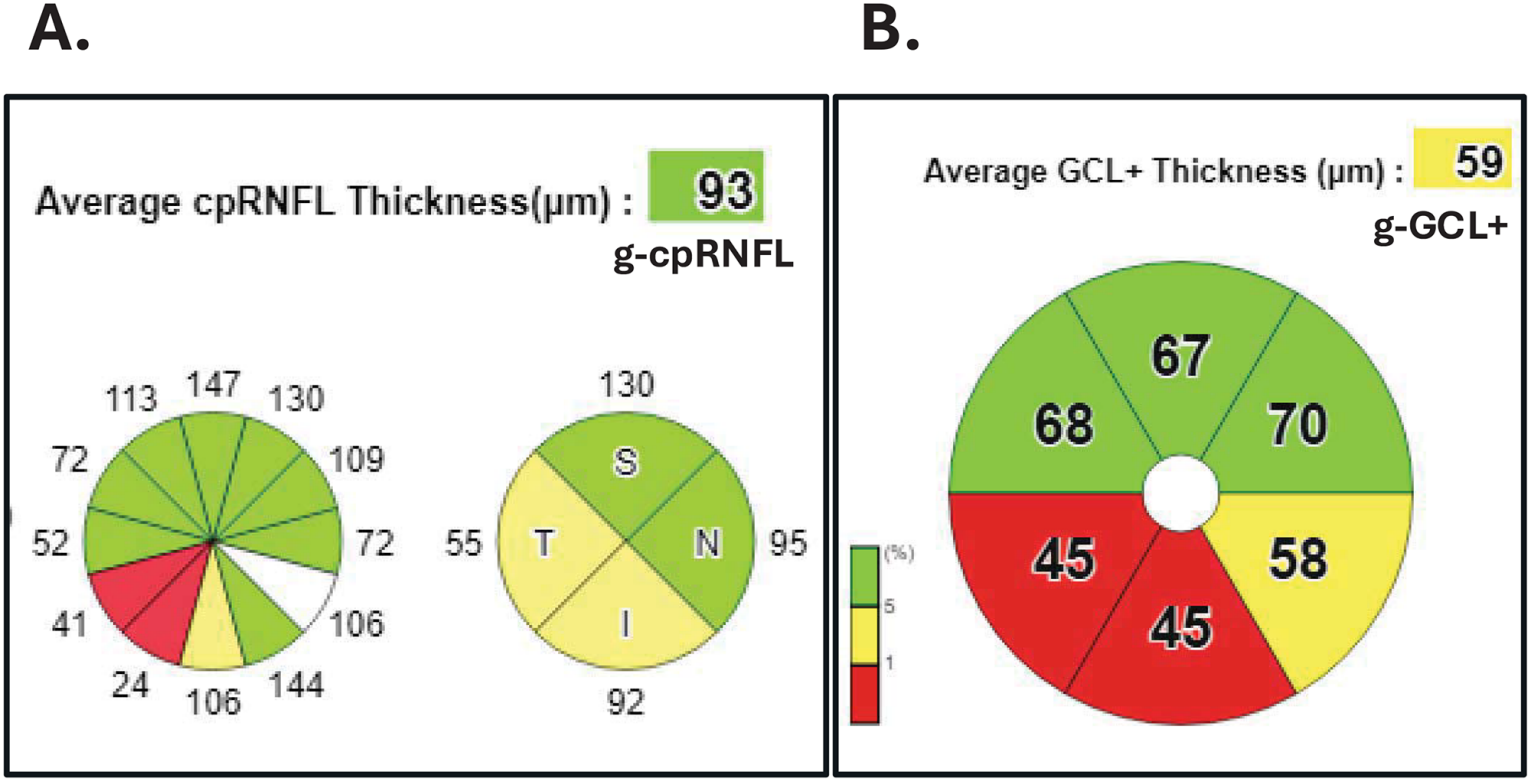
(**A**) Commercial representation of the average (global), clock hour, and quadrant thicknesses of the cpRNFL. (**B**) Commercial representation of the average (global) and sector thicknesses of the GCL+ thicknesses of the central 8°.

In OCT reports, these metrics are color-coded to flag those values falling below the lower fifth (yellow) or first (red) percentile of a reference database (RDB) of healthy control individuals. Figure 1A, from a commercial report, shows a typical display of the cpRNFL thickness and the associated percentile level for the global (g-) average, clock hours, and quadrants around the disc. Figure 1B shows similar information for the global average and the sectors of the g-GCL+ thickness within 10° of fixation.

The clinical utility of the metrics in Figure 1 and their associated color flags depends upon the RDB on which they are based. These RDBs are expensive and time-consuming to develop, as they typically involve a study with multiple sites and multiple tests (e.g., fundus exams/photographs, visual fields, OCT scans). Consequently, the commercial RDBs are relatively small. For example, there are 398 eyes in the commercial (C-) RDB underlying the summary statistics in Figure 1, and the RDBs in other commercial OCT instruments currently range from approximately 300 to 850 individuals.^4^^–^^6^

The size of these C-RDBs creates problems. First, if one can assume that the OCT metric in question (e.g., cpRNFL thickness) is described by a Gaussian distribution with equal variance at all ages, then linear regression can be used to set the fifth and first percentile cutoffs as a function of age. However, without a large sample from the target population, these assumptions are questionable. To avoid the Gaussian assumptions, quantile regression can be employed^4^^,^^5^ and has been used in at least two OCT devices recently cleared by the US Food and Drug Administration (FDA).^6^^,^^7^ Second, as we previously showed, the 95% confidence interval (CI) around the fifth and first cutoff values of the 398-eye C-RDB was so large that it nearly included the 50th percentile (median) values of these metrics. As expected, the 95% CIs for an RDB of 4.9K eyes were considerably smaller.^8^ This larger real-world (RW)–RDB was created by identifying healthy control eyes tested at optometry sites using a reading center method based on only OCT reports.^8^^,^^9^ The anatomic characteristics of this RW-RDB were similar to those of the existing commercial 398-eye RDB.^7^ Although the RW-RDB had greater precision (e.g., smaller CIs), this does not mean it will improve clinical decisions.

In this study, we compared the healthy control and glaucoma eyes flagged as within normal limits (green) or outside normal limits (yellow or red) by the C-RDB with 398 eyes to those flagged by the larger RW-RDB. The first and fifth percentile limits of the two RDBs were compared to a Gaussian model and Monte Carlo simulations.

## Methods

### OCT Scans

Widefield (12 × 9 mm) OCT scans (Maestro OCT; Topcon, Tokyo, Japan) were obtained from all participants. All OCT metrics, which included g-cpRNFL and g-GCL+ thickness, as well as cp-RNFL quadrant and clock hour and GCL+ sector thicknesses, were automatically generated without alteration of the software-generated segmentation or disc and foveal centering.

### The Reference Databases (RDBs)

#### Existing C-RDB

The C-RDB in the Maestro OCT instrument has 398 eyes from 398 individuals. As described in Chaglasian et al.,^6^ these eyes were categorized as “healthy” based on a complete ophthalmologic exam and a visual field test. Both eyes were free of ocular pathology, except for cataracts, and had visual acuity of 20/40 or better and intraocular pressure (IOP) ≤21 mm Hg. Refractive error was not an exclusion criterion. Their mean age was 46.2 ± 16.3 years (range, 18 to 88), and 57% were female. The means for the 398 g-cpRNFL and g-GCL+ thicknesses were 104.7 ± 11.9 (range, 67.2 to 134.3) and 71.3 ± 6.0 (range, 53.8 to 89.2).

#### Large RW-RDB

The RW-RDB contained 4830 eyes from 4830 individuals. The 4830 eyes were categorized as “healthy” based on OCT as previously described.^8^^,^^9^ Their mean age was 48.6 ± 17.0 years (range, 18 to 90), and about 60% were female. The means for the 4830 g-cpRNFL and g-GCL+ thicknesses were 103.3 ± 10.9 µm (range, 63.1 to 143.6) and 71.1 ± 5.8 µm (range, 49.6 to 93.5), respectively. As with the C-RDB, refractive error was not an exclusion criterion.

The 4830 eyes/individuals are a subset of the 4932 eyes/individuals previously described.^8^ Based upon subsequent analysis, 102 individuals were removed. These 102 included duplicate records (same patient with different IDs in 55 cases), nasal artifacts (4), scan quality (20), missing data (14), and/or pathology (9). The anatomic parameters of the C-RDB with the RW-RDB are virtually indistinguishable from those previously published for the slightly larger 4923 RW-RDB.^8^

### Test Sets

#### Healthy Control (H) Eyes

The H test set consisted of 175 eyes from 175 individuals who were part of a previous study.^10^ These individuals were recruited for a visual field RDB, and OCT scans were acquired as ancillary information, making them more typical of what one expects from a clinic. The mean age was 49.3 years (range, 22.1 to 85.5), and 61% were female. The means for the 175 g-cpRNFL and g-GCL+ thicknesses were 104.3 ± 11.0 µm and 71.5 ± 5.6 µm, respectively. None of these 175 H eyes were part of either RDB.

#### Eyes With Optic Neuropathy Consistent With Glaucoma (ON-G) Defects

These 183 eyes from 183 individuals, tested in optometry practices, had arcuate defects consistent with glaucomatous optic neuropathy seen on OCT reports, as previously described.^13^ The defects ranged from subtle defects in one hemiretina, which could be missed with visual field testing, to large defects in both hemiretinas. Thus, they ranged from OCT suspects to advanced glaucoma. The mean age was 68.5 ± 7.5 (range, 60 to 92), and 57% were female. The means for the 183 g-cpRNFL and g-GCL+ thicknesses were 81.0 ± 16.5 µm and 61.3 ± 7.20 µm, respectively. These data were part of a retrospective study approved by the Advarra Institutional Review Board (IRB).

Receipt and analysis of all the data were approved by the IRB of Columbia University*.*

### Analysis

#### OCT Metrics

Our focus is on the two most common OCT metrics used in clinical trials, the g-cpRNFL and g-GCL+ thickness. The g-cpRNFL is taken as the average of the cpRNFL thickness of a circle 3.4 mm in diameter centered on the disc, while the g-GCL+ thickness is the average GCL+ thickness within an annulus with a 10° outer radius centered on the fovea.

In addition, 22 other metrics were calculated: the cpRNFL thickness for the four cpRNFL quadrants and 12 cpRNFL clock hours (Fig. 1A) and the GCL+ thickness for the six sectors of the central ±10° (Fig. 1B).

#### Quantile Regression

Quantile regression was employed to derive the fifth and first percentile quantile regression lines (QRLs) and associated cutoff thicknesses.^5^ To be consistent with the commercial software used to generate the thickness in Figure 1, quantile regression incorporated both disc area and age for all 17 cpRNFL thickness metrics, while for all 7 GCL+ thickness metrics, only age was used. For the estimation of the 95% confidence bands, we used a method that is based on inverting a rank test as proposed by Koenker.^11^ The core idea is to find a range of coefficient values for which a rank test statistic would not be significant. This approach is robust as it does not require assumptions about the distribution of the error term and works better when the sample size is less than 1000 (like the C-RDB).

#### The Gaussian Model

The prediction of a Gaussian model was compared to the actual QRLs. The predicted QRLs are derived by assuming that the thickness at all ages is normally distributed with the same variance estimated from all the data but with a mean that is given by the linear regression of the mean. Thus, the fifth and first percentile cutoff lines have the same slope as the linear regression of the mean and an intercept based on the *z*-scores of a Gaussian distribution with a constant variance.

#### Monte Carlo Simulations

To better understand the effects on the variability of QRLs obtained with a sample of 398 eyes from a larger population, we obtained samples of size 398 from the larger RW-RDB. The sampling distribution and confidence intervals of slopes and intercepts of QRLs were obtained via Monte Carlo simulations for all metrics of interest (i.e., the two global metrics and the other 22 GCL+ and cpRNFL sectors and clock hours). In each of 1000 iterations, a random sample of 398 eyes was drawn from the 4830 eyes in the RW-RDB without replacement. Slopes and intercepts for the fifth and first percentiles were calculated for each of the 1000 samples, based on quantile regression. Each simulation yielded an empirical distribution of 1000 estimates. The 95% confidence interval was determined directly from this distribution using the percentile method, defined by the 2.5th and 97.5th percentiles of the simulated outcomes.

## Results

### The QRLs for the C-RDB and RW-RDB Differ

**Figure 2.**
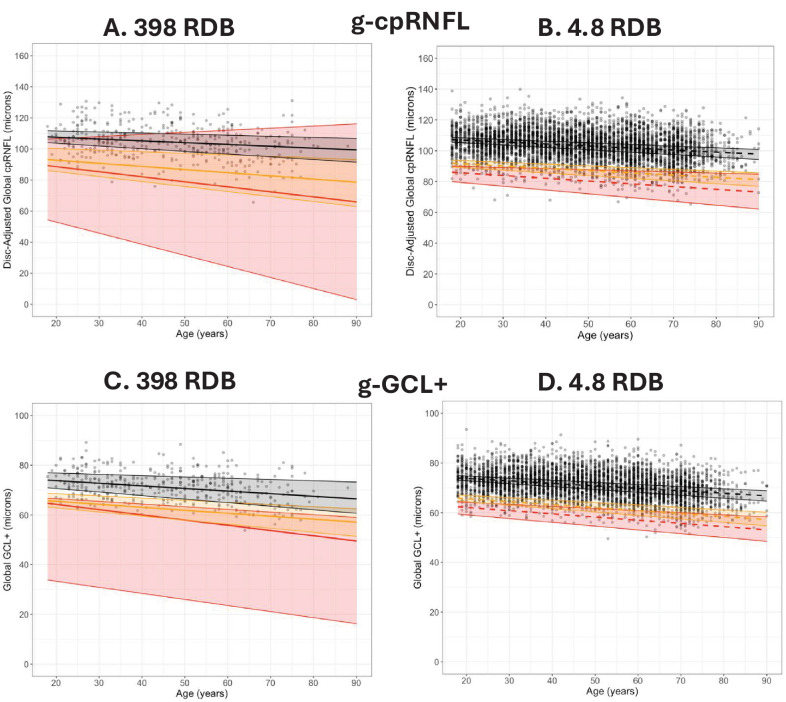
(**A**) The global (g-) cpRNFL thickness adjusted for disc area for the 398 eyes in the 398-eye C-RDB is plotted against age. The QRLs based on the 398-eye C-RDB are shown for the 50th (*black*), fifth (*yellow*), and first (*red*) percentiles. (**B**) The global (g-) cpRNFL thickness adjusted for disc area for the 4830 eyes in the 4830-eye RW-RDB is plotted against age. The QRLs based on the 4830-eye RW-RDB are shown for the 50th (*black*), fifth (*yellow*), and first (*red*) percentiles. (**C**) Same as panel A but for the g-GCL+ thickness. (**D**) Same as panel B but for the g-GCL+ thickness. In all panels, the *shaded regions* are the 95% CIs for the first (*red*), fifth (*orange*), and 50th (*black*) quantile regression cutoffs.

The data points in Figure 2A are the g-cpRNFL thickness for the 398 eyes in the C-RDB, plotted against age. The solid lines in Figure 2A are the QRLs for the fifth (yellow) and first (red) percentiles for this C-RDB. Figure 2B shows the same information for the 4830-eye RW-RDB. In both cases, g-cpRNFL thickness was adjusted for disc area, as with the commercial instrument. Figures 2C, 2D show the same representation of the g-GCL+ thickness. In this case, there was no adjustment due to the disc area, consistent with the commercial instrument. The shaded regions show the 95% CIs around the 50th (black), fifth (yellow), and first (red) QRLs.

**Figure 3.**
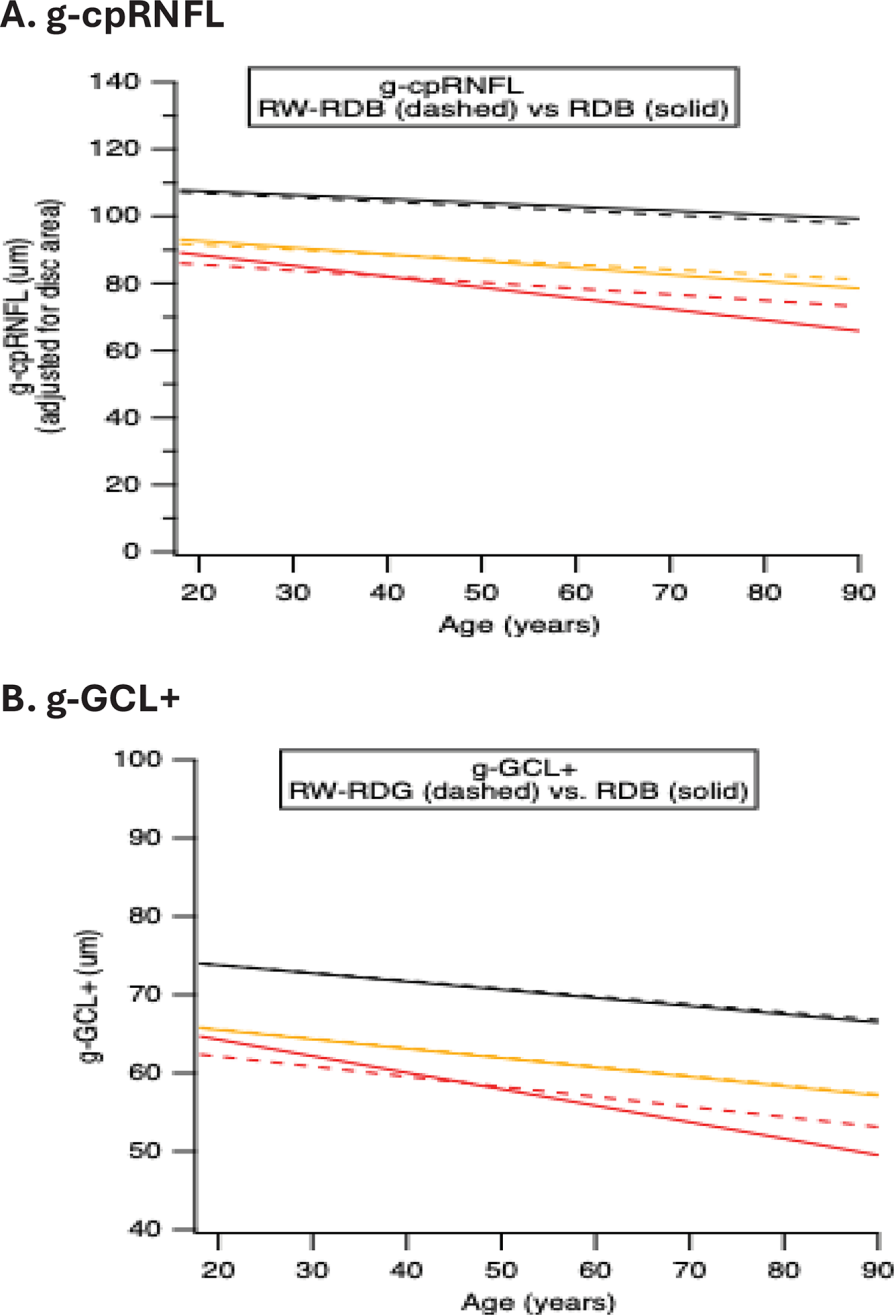
(**A**) A comparison of the C-RDB (*solid lines*) and RW-RDB QRLs (*dashed lines*) for the g-cpRNFL thickness from Figures 2A, 2B. (**B**) A comparison of the C-RDB (*solid lines*) and RW-RDB (*dashed lines*) QRLs for the g-GCL + thickness from Figures 2C, 2D. The QRLs are shown for the 50th (*black*), fifth (*yellow*), and first (*red*) percentiles.

Figure 3 shows a comparison of the QRLs (from Fig. 2) for the two RDBs for both the g-cpRNFL (Fig. 2A) and g-GCL+ (Fig. 2B) thickness. The QRLs are shown as solid lines for the C-RDB and dashed lines for the large RW-RDB. For both global metrics, the QRLs for the two RDBs are close for the 50th (black) and fifth (yellow) percentiles but markedly different for the first (red) percentile QRLs.

### The Two RDBs Do Not Flag an Identical Set of Eyes as Red or Yellow

#### The 175 H Eyes

**Figure 4.**
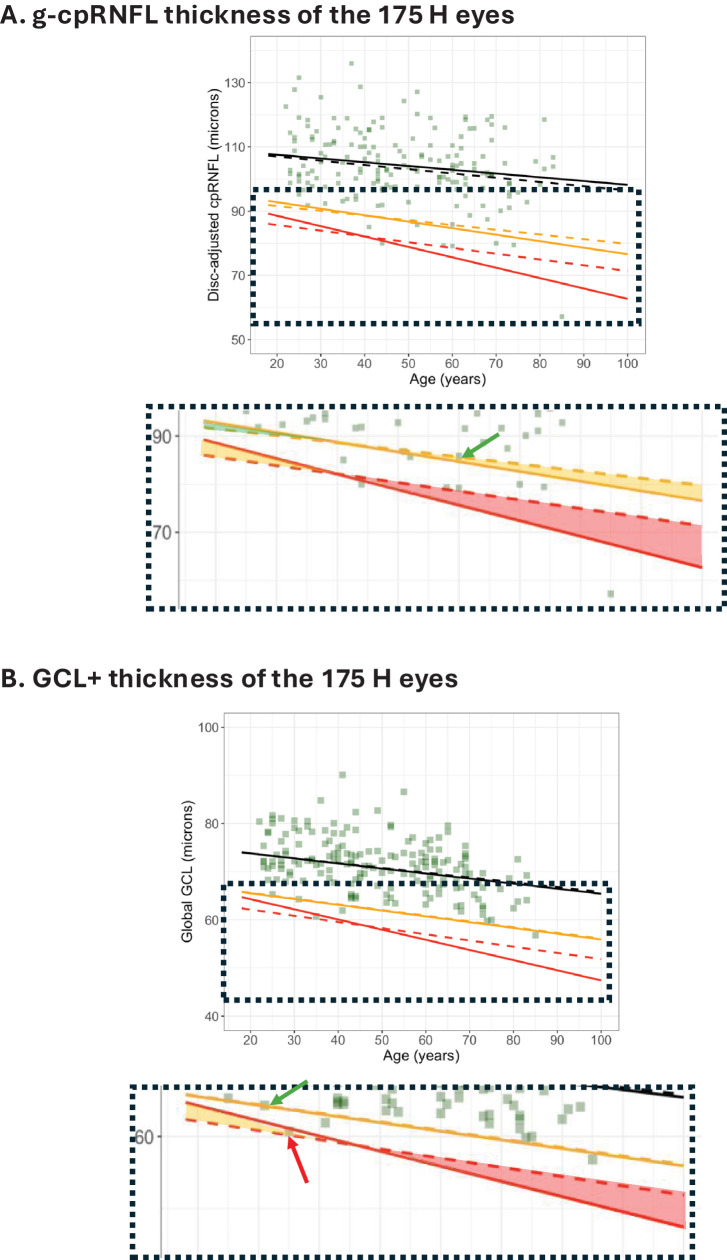
(**A**) The g-cpRNFL thickness adjusted for disc area for the 175 healthy eyes in the H cohort is shown with the QRLs from Figure 3A. The region within the *dashed rectangle* is expanded in the *inset* below the figure. The *shaded regions* indicate where the color flag for the two RDBs will differ. For example, the point with the *green arrow* will be coded green based on the C-RDB but *yellow* based on the RW-RDB. (**B**) The g-GCL+ thickness for the 175 healthy eyes in the H cohort is shown with the QRLs from Figure 3B. For example, the point with the *red arrow* will be coded *red* based on the C-RDB but *yellow* based on the RW-RDB.

The differences in slope and intercept of the QRLs for the two RDBs in Figure 3 imply that the eyes flagged as yellow or red will not necessarily be the same. In fact, if the thickness of an individual eye falls between the solid and dashed lines in Figure 3, then the flag will differ for the two RDBs. Figure 4 provides an illustration. The QRLs for the cpRNFL (Fig. 3A) and GCL+ (Fig. 3B) are shown with the 175 H eyes. The section of the graph within the dotted rectangle is enlarged and displayed. Only one data point falls between the QRLs for the C-RDB (solid) and the larger RW-RDB (dashed); it is indicated by the green arrow. This data point falls below the yellow dashed line but above the yellow solid line (i.e., in the yellow shaded region in Fig. 4A). Thus, this eye is flagged green based on the fifth percentile QRL of the C-RDB and yellow based on the RW-RDB. For the first percentile, none of the H eyes fell between the QRLs (red solid and dashed lines) for the two RDBs. The first column of Table 1 summarizes these results for the g-cpRNFL thickness. The flagged color changed for only one (0.6%) eye.

**Table 1.** Global cpRNFL and GCL+ Thickness for 175 H Eyes: Change in Color-Coding of 175 H Eyes When Switching From the 398-Eye RDB to the 4830-Eye RW-RDB

398→4830	g-cpRNFL	g-GCL+
G to Y	1	1
Y to G	0	0
Y to R	0	0
R to Y	0	1
Total	1 (0.6%)	2 (1.1%)
Change in FPs (specificity)		
5%	1 (−0.6%)	1 (−0.6%)
1%	0 (0%)	−1 (0.6%)

The second column of Table 1 contains the results for the g-GCL+ thickness. As can be seen in Figure 4A, although the fifth percentile QRLs are nearly identical, the green flag changed to yellow for 1 (0.6%) of the 175 eyes (green arrow). For the first percentile QRL, one (0.6%) eye changed from red to yellow (red arrow) with the change from the C-RDB to the RW-RDB. Overall, two (1.1%) eyes changed flag color for g-GCL+.

Table 1 also shows the change in the number of false positives (FPs). For the two global metrics and the 175 H eyes, the change in the number of FPs, and thus specificity, is relatively small. For the combination of first and fifth percentiles, the average (values) of change in sensitivity was 0.3% (0% and 0.6%) for the g-cpRNFL and 0% (−0.6% and 0.6%) for the g-GCL+ metric. Note that for the cells in the lower two rows in Table 1 as well as Supplementary Tables S1 to S3, red indicates a decrease in specificity and green indicates an increase in specificity based on the 175 H eyes, while for Table 2 and Supplementary Tables S4 to S6, red indicates a decrease in sensitivity and green indicates an increase in sensitivity based on the 183 ON-G eyes.

**Table 2.** Global cpRNFL and GCL+ Thickness of 183 ON-G Eyes: Change in Color-Coding When Switching From the 398-Eye RDB to the 4830-Eye RW-RDB

398→4830	g-cpRNFL	g-GCL+
G to Y	8	2
Y to G	0	0
Y to R	22	10
R to Y	0	0
Total	30 (16.4%)	12 (6.6%)
Change in the number of TPs (sensitivity)		
5%	8 (4.4%)	2 (1.1%)
1%	22 (12.0%)	10 (5.5%)

#### The 183 ON-G Eyes

**Figure 5.**
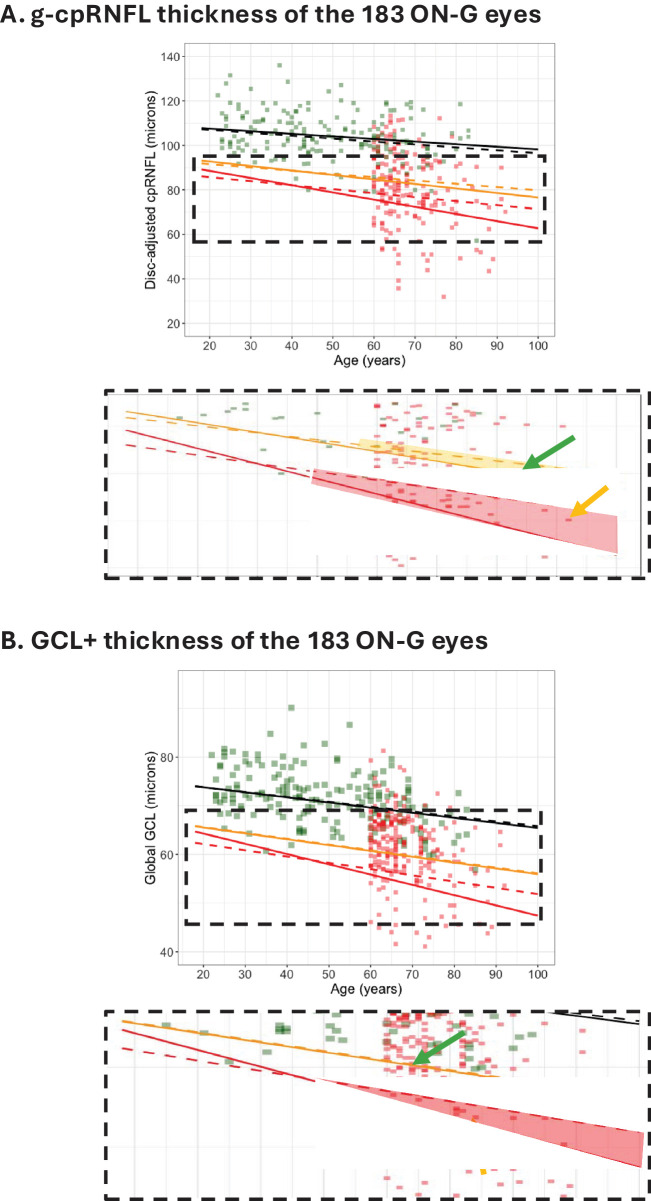
(**A**) The g-cpRNFL thickness adjusted for disc area for the 183 eyes in the ON-G cohort is shown with the QRLs from Figure 3A. The region within the *dashed rectangle* is expanded in the *inset* below the figure. The *shaded regions* indicate where the color flag for the two RDBs will differ. A point falling within the *yellow region* (e.g., the point indicated by the *green arrow*) will be coded *green* based on the C-RDB but *yellow* based on the RW-RDB, and a point falling within the *red region* (e.g., the point indicated by the *yellow arrow*) will be coded *yellow* based on the C-RDB but *red* based on the RW-RDB. (**B**) The g-GCL+ thickness adjusted for disc area for the 183 eyes in the ON-G is shown with the QRLs from Figure 3B. A point falling within the *red region* (e.g., the point indicated by the *yellow arrow*) will be coded *yellow* based on the C-RDB but *red* based on the RW-RDB. The *green arrow* points to an eye coded *green* based on the C-RDB but *yellow* based on the RW-RDB.

The number of eyes in the ON-G cohort that changed is considerably larger than the number that changed for the 175 H eyes. This is illustrated in Figure 5, where the data for the 183 glaucoma eyes (red symbols) are added to the figures in Figure 4.

Again, eyes falling between the cutoffs of the two RDBs will have different color flags for each RDB. In Figure 5A, 22 eyes fall in the red shaded region. The yellow arrow in the lower inset points to one of them. The flags for the cpRNFL thickness of these 22 eyes changed from yellow to red. Similarly, 8 eyes fall in the yellow shaded region (green arrow points to one) of the lower inset of Figure 5A. The flags for the cpRNFL thickness of these eyes changed from green to yellow. Overall, the color flag changed in 30 (16.4%) of the 183 eyes, shifting from the 398-eye C-RDB to the 4830-eye RW-RDB. These results are shown in the first column of Table 2.

The second column of Table 2 shows the results for the GCL+ thickness. Overall, a total of 12 (6.6%) changed flag color, 2 went from green to yellow (the green arrow points to one of them), and 10 went from yellow to red (the yellow arrow points to one of them).

For the combination of the first and fifth percentiles, the average (values) of change in sensitivity was 8.2% (4.4% and 12.0%) for the g-cpRNFL and 3.3% (1.1% and 5.5%) for the g-GCL+ metric.

#### Other Metrics

Supplementary Tables S1 to S6 contain the same information as in Tables 1 and 2, but in this case, for the cpRNFL thickness of the four quadrants and 12 clock hours (see Fig. 1A) and for the GCL+ thickness of the six sectors of macula (Fig. 1B). The results for these smaller regions of the cpRNFL and GCL+ regions were similar to those for the global metrics. For example, for the 175 H eyes (Supplementary Tables S1–S3), the number of eyes with a change in flag color was smaller than for the 183 ON-G eyes (Supplementary Tables S4–S6). For the combination of the first and fifth percentiles (fifth row total of tables), the average (range) percentage of the 175 H eyes changing color was 1.6% (0.6% to 2.9%) for the four quadrants, 1.5% (0% to 3.4%) for the 12 clock hours, and 1.2% (0% to 2.9%) for the six GCL+ sectors. On the other hand, for the combination of the first and fifth percentiles, the average (range) percentage of the 183 ON-G eyes changing color was 10.9% (1.6% to 21.9%) for the four quadrants, 18.8% (0.5% to 39.3%) for the 12 clock hours, and 11.7% (2.7% to 20.2%) for the six GCL+ sectors.

### Effects of Sample Size

To explore the hypothesis that the differences between the two RDBs are largely due to sample size, we performed two analyses.

#### The Gaussian Model

**Figure 6.**
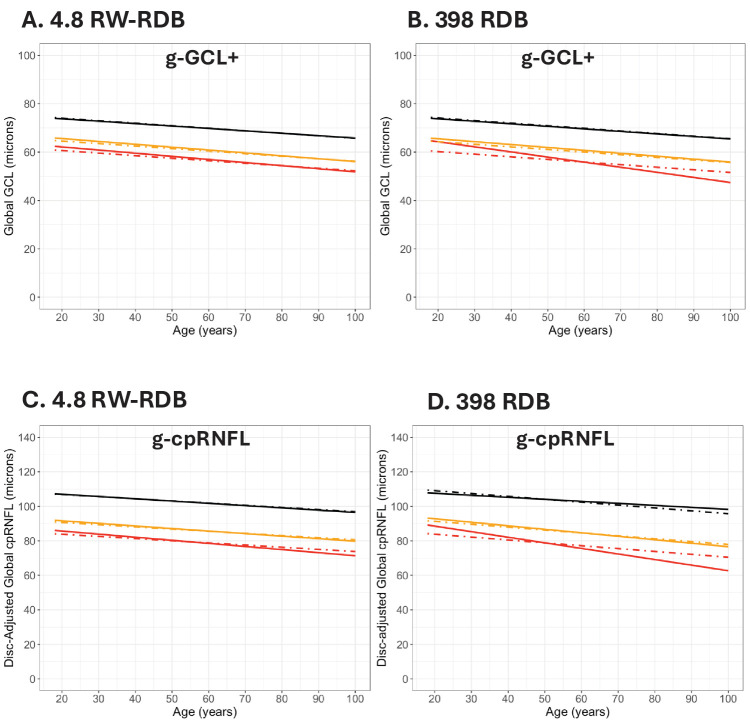
A comparison of the fifth percentile (*yellow*) and first percentile (*red*) QRLs (*solid lines*) to predictions from the Gaussian model (*dot dashed lines*) is shown for the g-GCL+ thickness and the RW-RDB (**A**) and C-RDB (**B**) and the g-cpRNFL thickness for the RW-RDB (**C**) and C-RDB (**D**).

To test the hypothesis that the larger RW-RDB will produce better estimates of the QRL, we compared each QRL with the predicted QRLs from a Gaussian model, as described in the Methods. Figures 6A, 6B show the QRLs for g-GCL+ thickness from Figure 3B for the RW-RDB (Fig. 6A) and C-RDB (Fig. 6B). The QRLs (solid lines) are compared to the predicted cutoff lines (dot dashed) of the Gaussian model. For the g-GCL+ thickness (Figs. 6A, 6B), the results for the fifth percentile (yellow) closely approximate those of the model for both RDBs (Figs. 6A, 6B). However, for the first percentile QRL, while the results for the RW-RDB are reasonably close (red lines in Fig. 6A), the results for the C-RDB deviate markedly for the first percentile (red lines in Fig. 6B).

The results for the g-cpRNFL in Figures 6C, 6D are similar. The Gaussian model provides a better fit to the RW-RDB than it does for the smaller C-RDB. In particular, although the RW-RDB shows a small difference in slope between the QRL and the Gaussian model, the difference is larger for the C-RDB. The results for the 22 local metrics (i.e., the four quadrants and 12 clock hours of the cpRNFL thickness and the six sectors of the GCL+ thickness in Supplementary Figs. S1–S3) agree. The Gaussian model fitted the RW-RDB better than it fitted the C-RDB. However, even for the RW-RDB, the QRLs (solid lines) deviated noticeably from the predicted Gaussian cutoffs at more localized metrics, like the cpRNFL clock hours.

#### Monte Carlo Simulations

**Figure 7.**
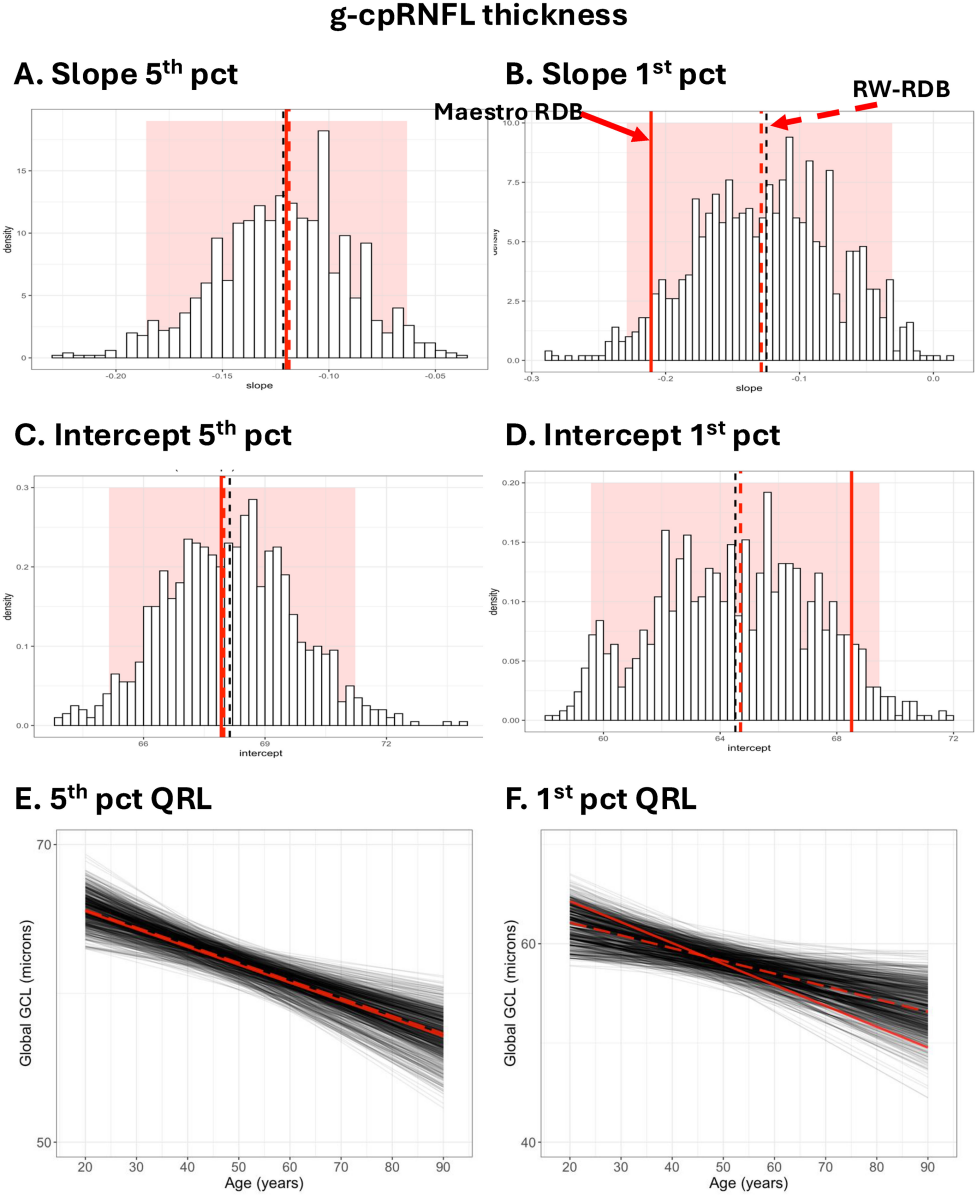
(**A**, **B**) For the g-cpRNFL thickness, the distribution of slopes for the fifth (**A**) and first (**B**) percentile QRLs of 1000 samples of 398 eyes from the 4830 individuals in the RW-RDB. (**C**, **D**) Same as A and B but for the intercept of the QRLs for the fifth (**C**) and first (**D**) percentile QRLs. (**E**, **F**) The fifth (**E**) and first (**F**) QRLs for the 1000 samples. In all panels, the *solid red line* represents the results for the 398-eye C-RDB, while the *dashed red line* represents the results for the RW-RDB. The *dashed black lines* in A to D are the means of the simulations.

As a proof of concept that the deviations of the 398-eye C-RDB QRLs from both the Gaussian model and the RW-RDB QRLs could be due to sample size, we performed Monte Carlo simulations. As described in the Methods, we obtained 1000 random samples of 398 eyes from the 4830-eye RW-RDB. Figures 7A–D show the results in the form of histograms for the slopes and intercepts for g-GCL+ thickness. The red vertical dashed lines are the values for the entire RW-RDB, which are close to the mean of the simulated distribution of samples of size 398 (black vertical dashed lines), as expected. The solid vertical red lines show the slopes and intercepts for the 398-eye C-RDB. For the fifth percentile (Figs. 7A, 7C), the solid red lines are very close to the red and black dashed lines. On the other hand, for the first percentile (Figs. 7B, 7D), the slope and intercept for the C-RDB (red solid lines) deviate from the slope and intercept for the RW-RDB (red dashed lines). However, the solid red vertical lines are well within the 95% confidence interval (red rectangle) of the distribution of simulated values. The QRLs for all 1000 samples in Figures 7E and 7F illustrate the same points. In any case, the simulations for samples of 398 individuals taken from the 4830-eye RW-RDB produce QRLs that are more extreme than the difference between the actual 398-eye C-RDB and the RW-RDB.

**Figure 8.**
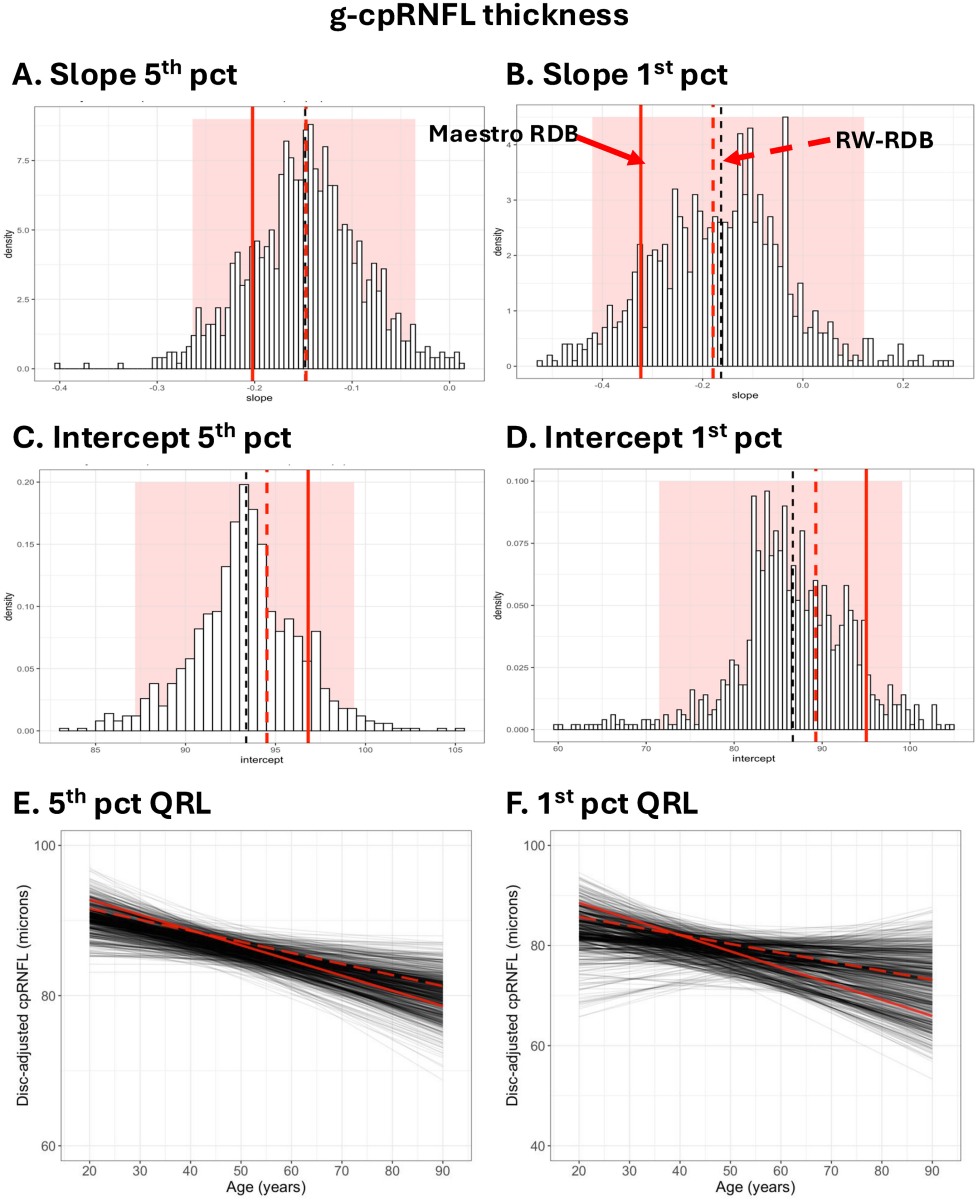
Same as Figure 7 but for the g-GCL+ thickness.

Figure 8 contains the same analysis for the g-cpRNFL thickness corrected for disc area. The conclusions are the same, although for g-cpRNFL, the RW-QRLs (red solid) for both the fifth and first percentiles deviate.

#### Other Metrics

The results for both the fifth and first percentiles for the 22 localized metrics of the cpRNFL and GCL+ are consistent. First, the simulations for samples of 398 individuals taken from the 4830-eye RW-RDB produce QRLs more extreme than the difference between the actual 398-eye C-RDB and the RW-RDB, as can be seen in Supplementary Figures S4 to S6, which are like the bottom panels for Figures 7 and 8. Second, 40 of the 44 slopes and 42 of the 44 intercepts for the 398-eye C-RDB fall within the 95% confidence limits of the simulations.

## Discussion

In this study, we compared the number of healthy control and glaucoma eyes flagged as outside normal limits (yellow or red) by the C-RDB with 398 eyes to those flagged by the larger RW-RDB with 4830 eyes. First, although most eyes are flagged the same, the results indicate that the C-RDB and the larger RW-RDB do not flag an identical set of eyes as red or yellow. Second, a Gaussian model fits the QRLs of the RW-RDB better than those of the smaller C-RDB. Third, the deviations of the C-RDB QRLs from both the Gaussian model's predictions and the larger RW-RDB QRLs can be mimicked by taking samples of 398 eyes from the RW-RDB. We expand upon these three points below and discuss the implications for the sensitivity and specificity of a large RW-RDB, as well as its potential to improve clinical decisions based on metrics.

### A Comparison of the Flagging by the Two RDBs

*The C-RDB and the larger RW-RDB do not flag an identical set of eyes as red or yellow.* Further, the percentage of the 183 ON-G/S eyes flagged differently was considerably larger than the percentage of the 175 H eyes flagged differently. For the g-cpRNFL metric, the flagging by the two RDBs differed by only 0.6% for the 175 healthy control eyes, while 16.4% of the 183 ON-G eyes were flagged differently.

The increase in the size of the RDB clearly affected the flagging of the ON-G/S eyes more than it did the H eyes. This difference should be expected when the specificity is set high (e.g., 95% or 99%) and the associated sensitivity is considerably lower than the specificity, as is the case for the current OCT metrics.

### The Gaussian Model

The Gaussian model was used as a “null model.” That is, we did not necessarily expect this model to accurately represent the underlying population of interest. We did, however, predict that the larger RW-RDB would better approximate this model than the small C-RDB. The Gaussian model predicts that the fifth and first percentile QRLs should be parallel and separated by a fixed amount that depends on the *z*-scores for the fifth and first percentiles. The QRLs for the RW-RDB fall close to those predicted by the model. While the small deviations between expected and observed results (Fig. 6, left column) could be due to random variation for the RW-RDB, they are also consistent with a small increase in variability of the metrics in older eyes, as opposed to younger eyes. Recall that the Gaussian model assumes that the variance of the metrics does not change with age.

In any case, the model provides a good description of the RW-RDB QRLs (Fig. 6, left column). On the other hand, the first percentile QRLs for the C-RDB markedly deviate from parallel, as seen in Figure 6 (right column). This is also obvious in Supplementary Figures S1 to S3, which contain the results for the other, more local 22 metrics. However, for some of the smaller regions of the cpRNFL (i.e., the quadrants and clock hours), the QRLs for the RW-RDB also clearly deviate from the model, raising the possibility that the model may yield more “accurate” flagging under these conditions.

### The Simulations of the Effects of an RDB of 398 Eyes

To simulate the effects on the QRLs of obtaining a sample of 398 eyes from a larger population, we obtained the distribution of the slopes and intercepts for 1000 samples from the 4830-eye RW-RDB. The slope and intercept of the fifth and first percentile QRLs of the actual 398-eye C-RDB fell within the 95% confidence limits of the distribution of QRLs for both the g-cpRNFL and g-GCL+ metrics for both the fifth and first percentile QRLs. This was also the case for 18 of the other 22 metrics.

### The Differences in the QRLs of the Two RDBs Are Due Largely to Sample Size

The RW-RDB and the C-RDB do not flag identical eyes. While increasing the size of a reference database (RDB) may or may not improve sensitivity, it is expected to enhance the accuracy of flagging, particularly if both RDBs are derived from the same statistical population and/or if the RW-RDB better reflects the target population. The latter is supported by the fact that the RW-RDB comes from a population screened for diseases such as glaucoma. However, the evidence also suggests that the two RDBs come from essentially the same statistical population. By this, we mean that the differences in the flagging by the RDBs are largely due to random variation, not systematic differences. There are three lines of evidence for this conclusion.

First, we previously compared seven anatomic parameters of the two RDBs and found that differences between the two RDBs, when present, were minimal.^8^

Second, the comparison of the QRLs of the RW-RDB to the Gaussian model is also consistent with the hypothesis that the differences in the flagging of the metrics by the RDBs are due to random variation, not systematic differences.

Third, the Monte Carlo simulations provide a proof of concept that the sample size of 398 from a larger population, in this case the 4830-eye RW-RDB, can yield QRLs with slopes and intercepts that deviate as much as or more than the slopes and intercepts of the 398-eye C-RDB deviate from the values for the 4830-eye RW-RDB or the Gaussian model.

Thus, overall, the results are consistent with the conclusion that the large RW-RDB will provide more accurate flagging of the OCT metrics and thus should improve clinical decisions based on these metrics.

### What Do We Mean by More Accurate Flagging?

There are two ways in which the flagging by the 4830-individual RW-RDB is more accurate than the existing 398-eye C-RDB. First, based on the test datasets used and the traditional meaning of test accuracy (i.e., total true positives [TPs] plus true negatives [TNs] divided by total individuals), the accuracy of the RW-RDB was 6.1% (g-cpRNFL) and 2.8% (g-GCL+) greater than the accuracy for the C-RDB for the first percentile QRL and 2.0% (g-cpRNFL) and 0.3% (g-GCL+) greater than the accuracy for the C-RDB for the fifth percentile QRL. In addition, based on the test datasets, while the change in specificity with RDB for the two global measures and the fifth and first percentile QRLs varied between −0.6% and +0.6%, the change in sensitivity was greater, especially for the first percentile QRL, where it was 6.1% (g-cpRNFL) and 2.8% (g-GCL+) greater (Tables 1, 2).

More important for the analyses in this study is the accuracy of the flagging provided by each RDB. Two RDBs may demonstrate similar overall test accuracy by the traditional metric (i.e., total number of TPs and TNs divided by total individuals) yet still flag individual eyes differently. In the current study, the flagging by the two RDBs of the 183 ON-G eyes differed by 16.4% for the g-cpRNFL, which was greater than the change in the number of total TPs + TNs, and the analysis above indicates the RW-RDB will produce more accurate flagging.

**Figure 9.**
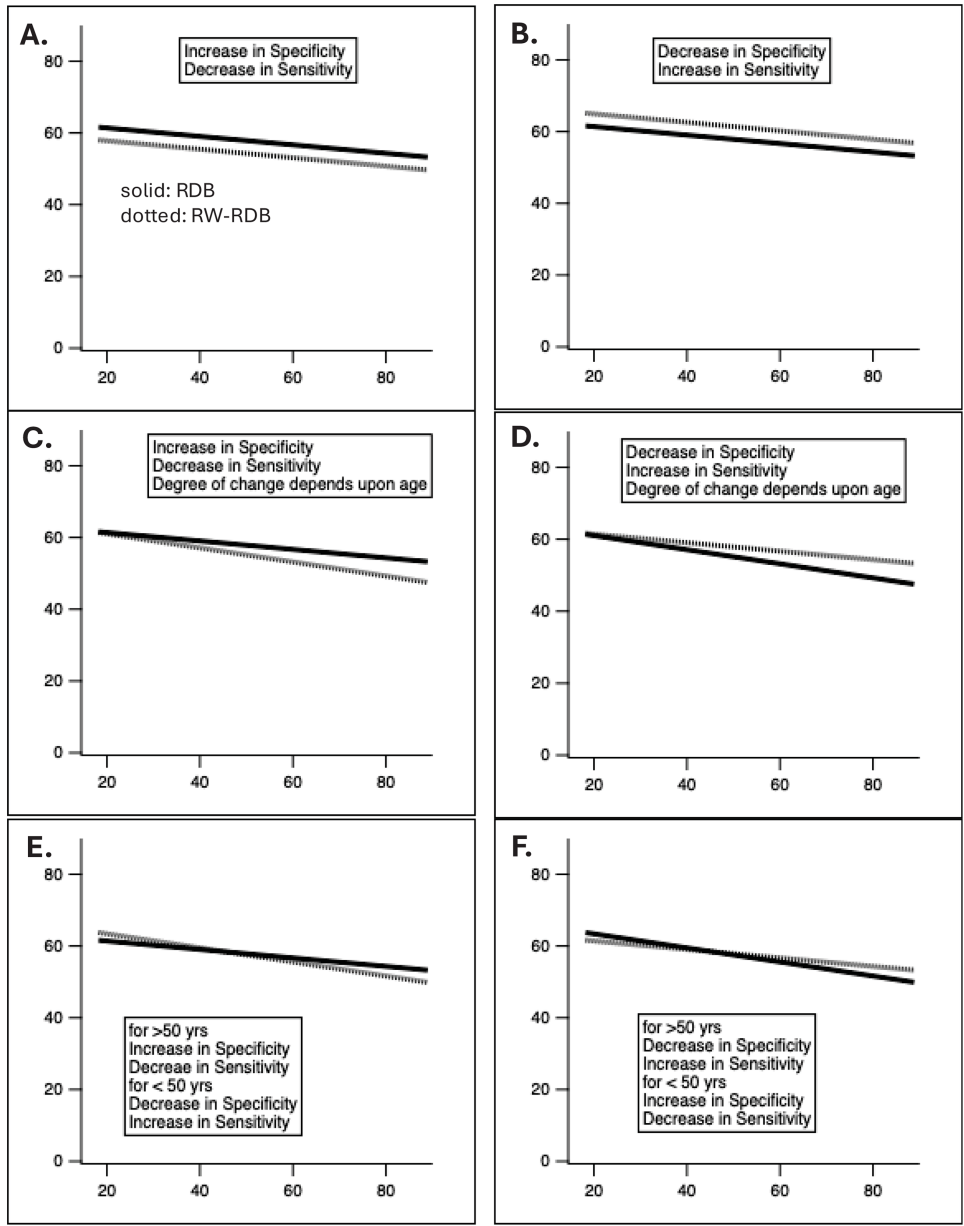
Hypothetical outcomes for the QRLs for changing from the C-RDB (*solid*) and RW-RDB (*dotted*) for a given cutoff percentile. (**A**, **B**) There could be a decrease (**A**) or an increase (**B**) at all ages. (**C**, **D**) There could be a decrease (**A**) or an increase (**B**), but the degree of change could be dependent upon age. (**E**) There could be a decrease in sensitivity but an increase in specificity for individuals above some age (e.g., >50 years) and an increase in sensitivity but a decrease in specificity for individuals below that age (e.g., <50 years). (**F**) There could be an increase in sensitivity but a decrease in specificity for individuals above some age (e.g., >50 years) and a decrease in sensitivity but an increase in specificity for individuals below that age (e.g., <50 years).

*Further, we want to be clear that increasing the size of* *an RDB* *should increase the accuracy of the flagging, but it will not necessarily increase sensitivity, specificity, or* *accuracy as traditionally measured.* In fact, theoretically, for a given age and metric, the larger RDB can increase sensitivity and decrease specificity or decrease sensitivity and increase specificity. To illustrate this point, Figure 9 illustrates six hypothetical situations for a given metric (e.g., g-cpRNFL) and a given percentile (e.g., first). The intercept and slope of the larger RW-RDB (dashed) can have better specificity but poorer sensitivity (Figs. 9A, 9C) or the reverse (Figs. 9B, 9D) at any given age, although the degree to which this is true varies with age in the case of Figures 9C and 9D. If the QRLs cross, as in Figures 9E and 9F, then the RW-RDB can increase specificity and decrease sensitivity for younger eyes but decrease specificity and increase sensitivity for older eyes (Fig. 9E) or the reverse (Fig. 9F). Examples of these hypothetical scenarios can be seen in Supplementary Figures S1 to S3.

Taken together, the evidence suggests that the larger RW-RDB will yield better clinical decisions. By “better clinical decisions,” we mean more accurate flagging of eyes as healthy controls or glaucoma, based on the fifth and first percentile QRLs. Take, for example, a hypothetical screening population of 1000 individuals with a 3% glaucoma prevalence (*N* = 30 cases). A 16.4% increase in sensitivity results in the detection of approximately five additional patients with glaucoma who would have otherwise been missed (flagged green/yellow instead of red). While these five individuals represent only 0.5% of the total population, they represent >16% of the disease population. In a screening context, correctly identifying 16% more of the pathology can be considered a substantial clinical improvement.

### Limitations and Caveats

There are three limitations, or at least caveats, worth mentioning. First, we are not claiming that the C-RDB and the RW-RDB come from identical populations. In fact, there are likely differences between these RDBs, as one comes from individuals recruited for clinical trials and screened with visual fields and clinical exams, while the RW-RDB comes from individuals examined in an optometry practice and OCTs screened by a reading center. One could argue that the former may contain “super normal” individuals, while the latter are more appropriate for the target population of the Maestro OCT. In any case, the evidence does suggest that, based on the metrics we have evaluated, the variation seen between the two RDBs is due largely to the relative sizes of these RDBs.

Second, as mentioned above, with increased levels of sensitivity, the difference in the number of eyes flagged by the two RDBs will decrease. In the extreme, if only eyes with advanced glaucoma were included, then the sensitivity would be 100% for both RDBs, and there would be no difference in flagging. One could argue that some of the 183 eyes are suspects and may not have glaucoma, and thus the sensitivity for the 183 eyes is artifactually low. However, we previously reported sensitivities of only 63% (cpRNFL) and 66% (g-GCL+) for a specificity of 96%,^12^ for a group of patients with early glaucoma (24-2 mean deviation (MD) better than –6 dB), confirmed with 24-2 and 10-2 visual fields. In any case, we chose the 183 ON-G for two reasons. One, it was collected under real-world conditions, and two, it had the full range of ON-G defects one would see in the clinic. In particular, the defects ranged from very early defects, which would be missed on visual fields and perhaps routine clinical exams, to advanced damage affecting both hemispheres and both macular regions.

Third, it could be argued that we are overstating the effect in a real-world situation. The poorer “accuracy of flagging” of the smaller sample was largely due to the change in flagging of the ON-G eyes, and these eyes would only be approximately 3% of a real-world population.^16^ However, the simulation results in Figure 8 suggest that a different sample of 398 could easily yield a larger error in the “accuracy of flagging” than observed here, while the alternative outcomes in Figure 9 illustrate how the accuracy of flagging could be relatively larger for the healthy cohort.

Fourth, by focusing on only one advantage of a larger RDB—namely, improved precision of metrics—we do not mean to imply it is the only reason to prefer the 4830-eye RW-RDB. First, the larger RW-RDB has allowed us to develop an improved metric using a logistical regression model.^14^ In addition to a better metric, with the larger sample size also come other advantages that may improve clinical decisions. We can now correct for other variables, which were not possible due to the lack of statistical power. For example, we know that fovea-to-disc distance has a larger effect on some of the metrics than does age or disc area, the two variables currently corrected for. Further, the larger RW-RDB has already allowed us to develop a deep learning model.^13^^,^^15^ Finally, it is often difficult to distinguish the lower end of normal and mild or early disease. With a C-RDB between about 300 and 850, there are only three to nine eyes in the lower 1% on any metric. The existing RW-RD has 48, and it should be relatively easy to expand this number in the future. Expanding will also allow for specialized or stratified RDBs such as for high myopes and/or ethnic groups.

Fifth, as we noted in a previous characterization of this database,^8^ the RW-RDB lacks specific metadata regarding race, ethnicity, and axial length. It is well established that cpRNFL thickness varies by race and is negatively correlated with axial length. Because these variables were not controlled for, the normative limits presented here represent a composite of the population visiting these specific clinical practices. Consequently, while this database offers improved statistical stability for this target population, the resulting limits may not be generalizable to populations with significantly different racial compositions or axial length distributions. On the other hand, in its present form, the RW-RDB allows us to control for axial length by using mirror position.^8^ Further, in the future, the data from real-world sites can potentially be combined with information about race/ethnicity and refractive error.

Finally, it is important to acknowledge a limitation inherent to the “accuracy of flagging” metric itself. The conventional green–yellow–red scheme relies on binary cutoffs (first and fifth percentiles), which can exaggerate small differences in RNFL thickness. For example, an eye flagged as yellow that changes to red may only change from the 1.1 to 1.0 percentile or, if changing from green to yellow, from the 5.1 to 5.0 percentile. While this study cannot resolve the limitations of binary classification, it demonstrates that a larger reference database reduces the sampling error around these rigid thresholds. This ensures that a change in flag color reflects a true difference in the population distribution rather than a spurious fluctuation caused by a small sample size. One possibility is to use a continuous color scheme like the one that already exists in the Maestro deviation/probability maps.

## Summary

In general, a larger reference database (RDB) derived from the same underlying population may or may not lead to an increase in the traditional accuracy metric (i.e., total true positives and true negatives divided by total individuals), but it will provide more accurate classification/flagging at the individual eye level. This is because the fifth and first percentile cutoffs will more precisely represent the true distribution of the target population.

These findings have implications for both the specific 398-eye C-RDB in this study, as well as the relatively small number of eyes in commercial RDBs, in general. In the specific case of replacing the 398-eye C-RDB with the 4830-eye RW-RDB, we conclude that the accuracy of flagging is improved with the larger RW-RDB. This improvement stems primarily from the increased sample size rather than any meaningful difference in the populations sampled. Moreover, based on the test sets evaluated, the RW-RDB demonstrated higher overall accuracy and sensitivity for the global metrics, with only a modest change in specificity.

## Supplementary Material

Supplement 1

Supplement 2

Supplement 3

Supplement 4

Supplement 5

Supplement 6

Supplement 7
