# Supplementary material for: Larger Real-World OCT Reference Database Improves Accuracy of Glaucoma Flagging Using Summary Metrics
Source: Transl Vis Sci Technol. 2026 Mar 9;15(3):6. doi: 10.1167/tvst.15.3.6 (PMC12988682; doi:10.1167/tvst.15.3.6)
Supplement: Supplement 1 [file tvst-15-3-6_s001.docx]

**
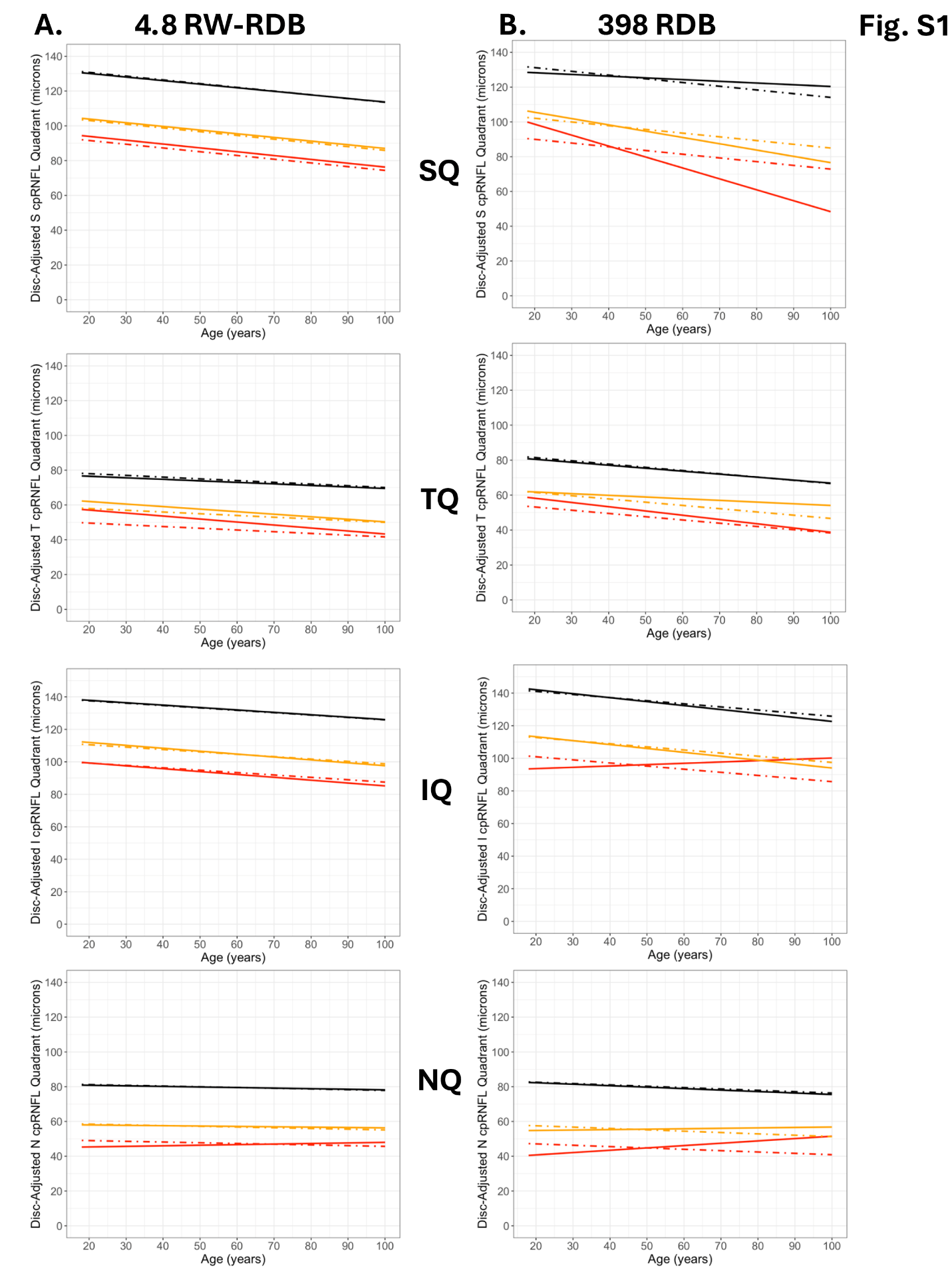
**

**Figure S1.** A comparison of the 5^th^ pct (yellow) and 1^st^ pct (red) QRLs (solid lines) to predictions from the Gaussian model (dot dashed lines) are shown for the disc area adjusted cpRNFL thickness of the 4 cpRNFL quadrants and the RW-RDB (A) and C-RDB (B).

**
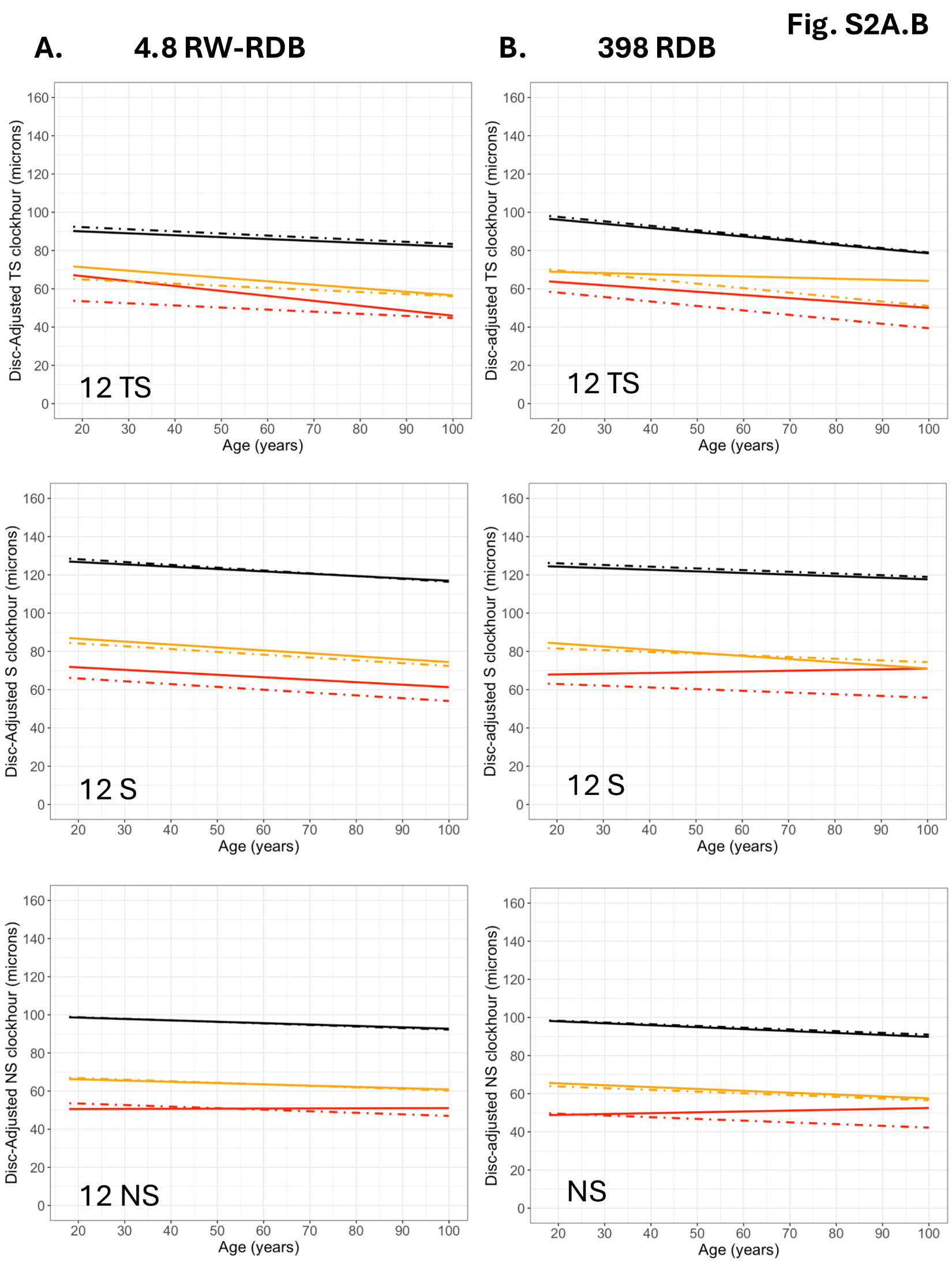
**

**
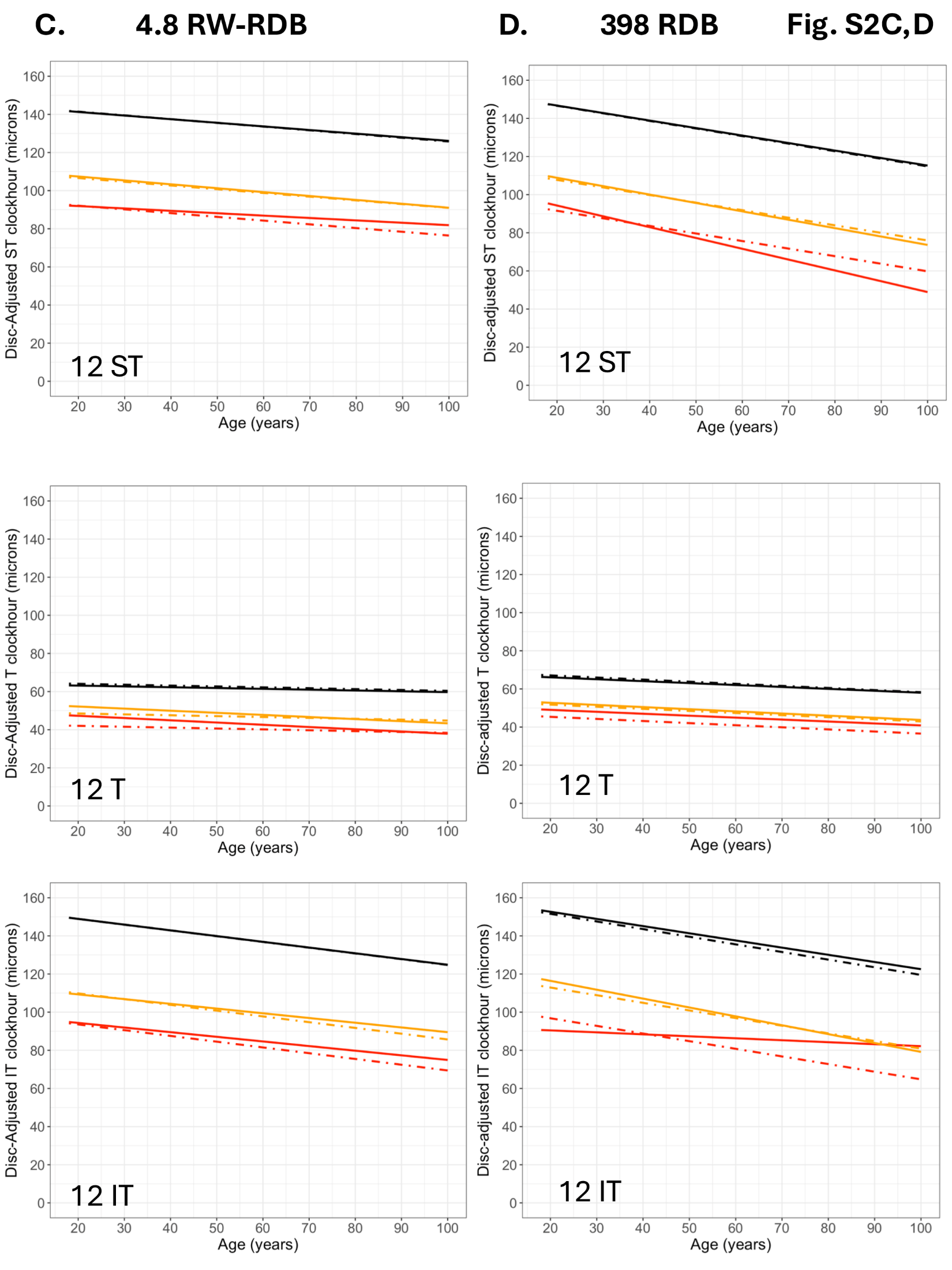
**

**
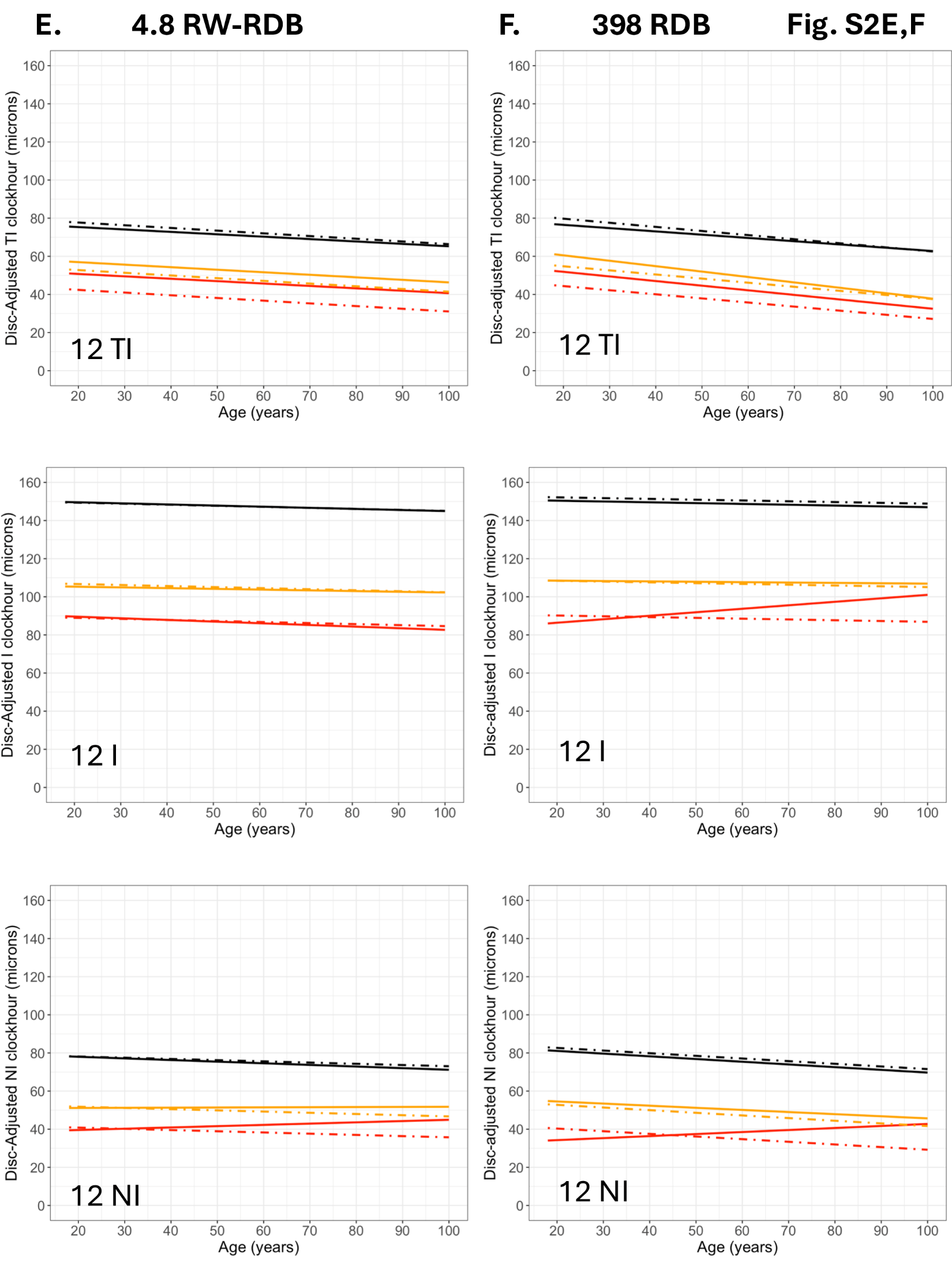
**

**
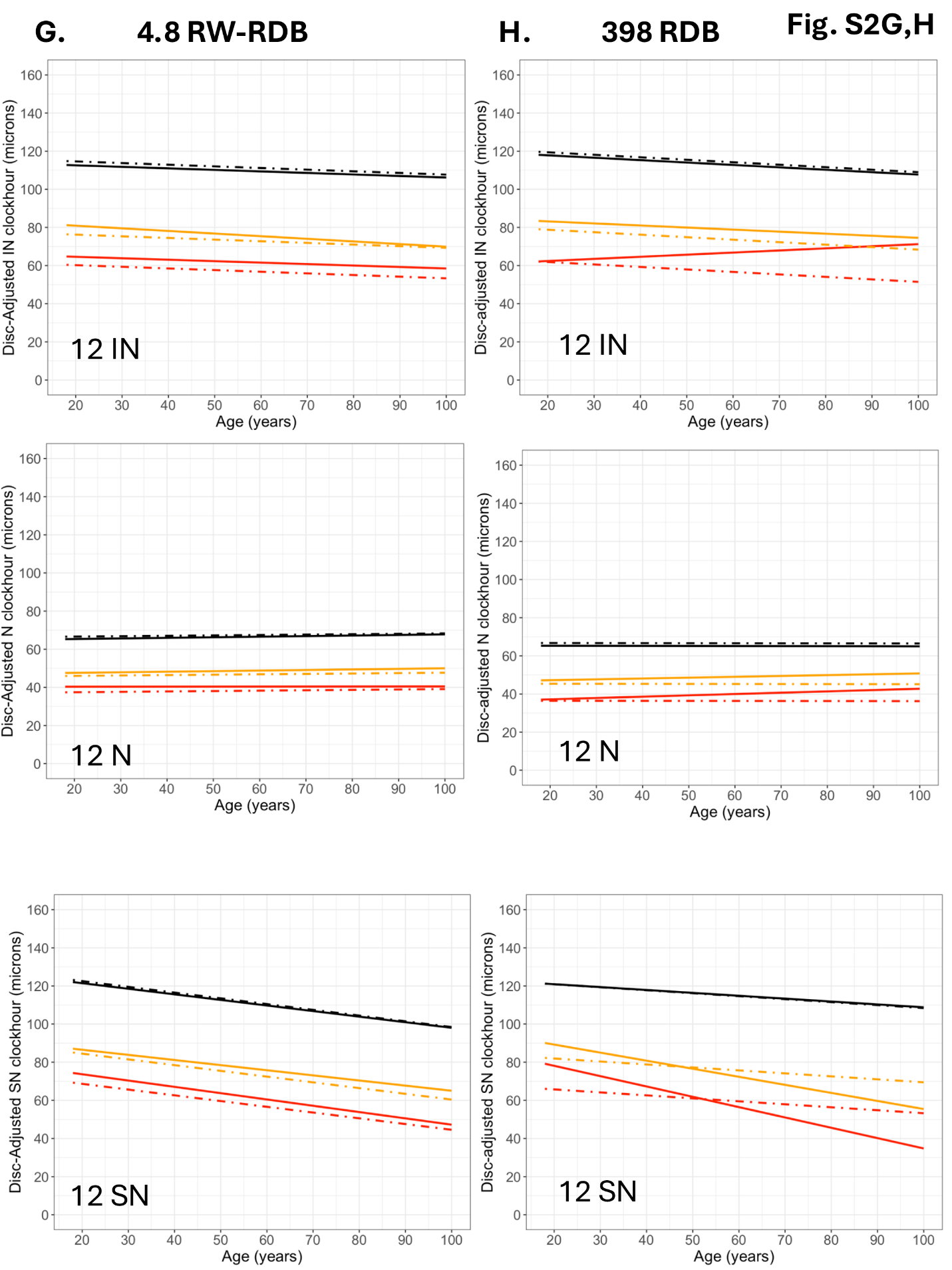
**

**Figure S2.** A comparison of the 5^th^ pct (yellow) and 1^st^ pct (red) QRLs (solid lines) to predictions from the Gaussian model (dot dashed lines) are shown for the disc area adjusted cpRNFL thickness of the 12 cpRNFL clock hours and the RW-RDB (A,C,E,G) and C-RDB (B,D,F,H).

**
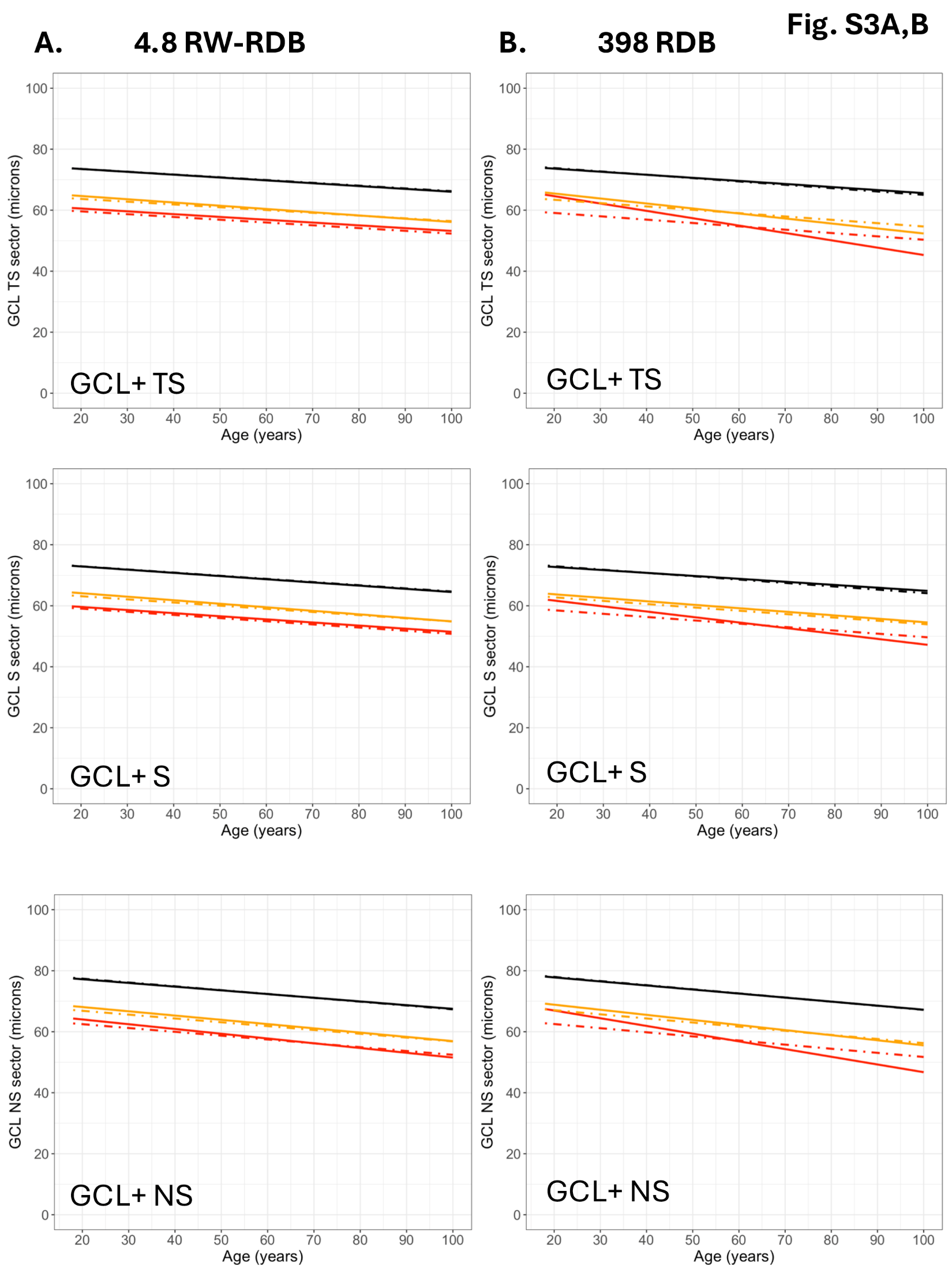
**

**
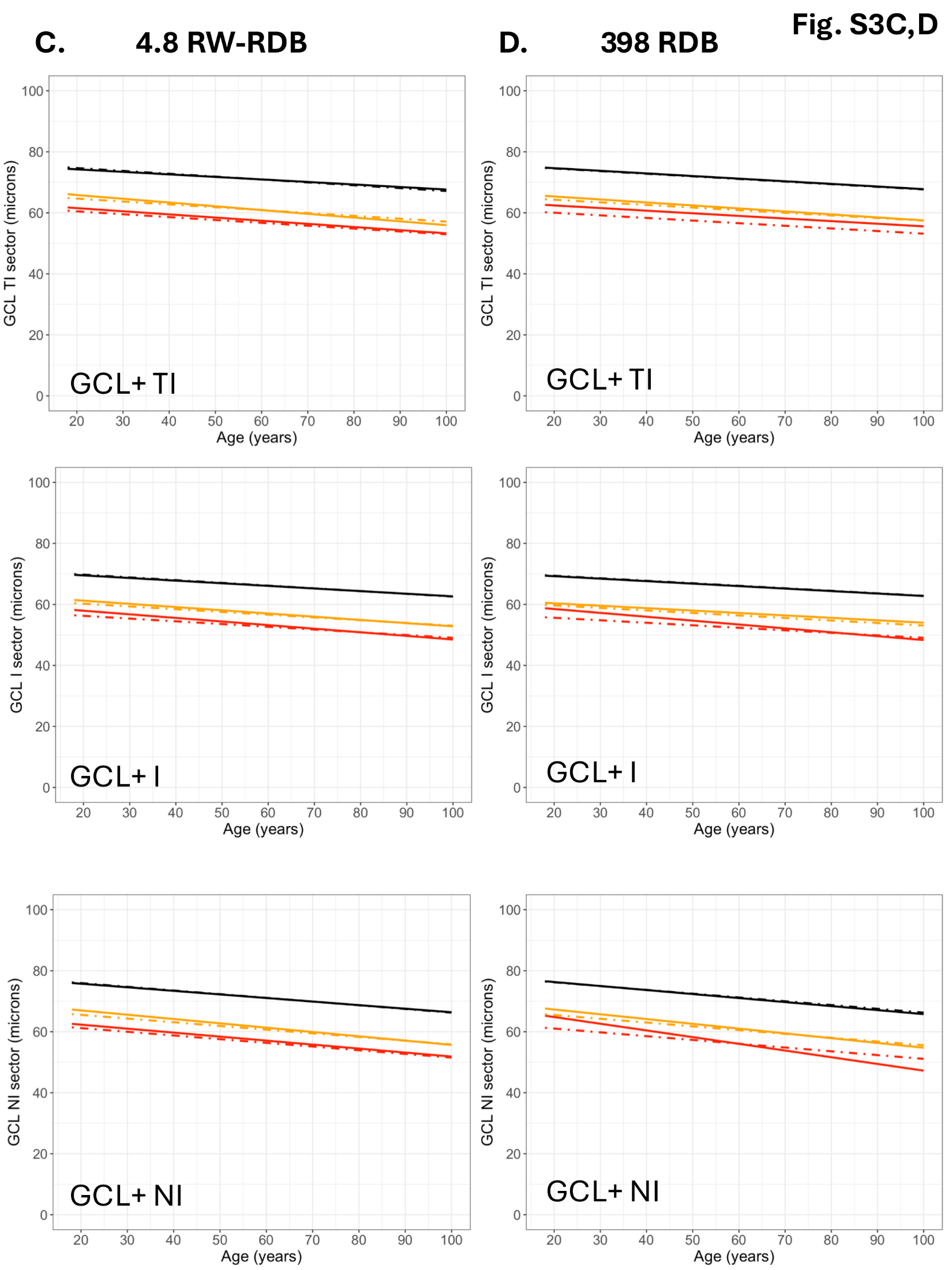
**

**Figure S3.** A comparison of the 5^th^ pct (yellow) and 1^st^ pct (red) QRLs (solid lines) to predictions from the Gaussian model (dot dashed lines) are shown for the GCL+ thickness of the 6 GCL sectors and the RW-RDB (A,C) and C-RDB (B,D,).

The thin black lines are the 5^th^ (left) and 1^st^ (right) pct QRLs for the 1000 samples of the 4 quadrants of the disc adjusted cpRNFL thickness. In all panels the solid red line represents the results for the 398 C-RDB, while the dashed red line represents the results for the RW-RDB.

**
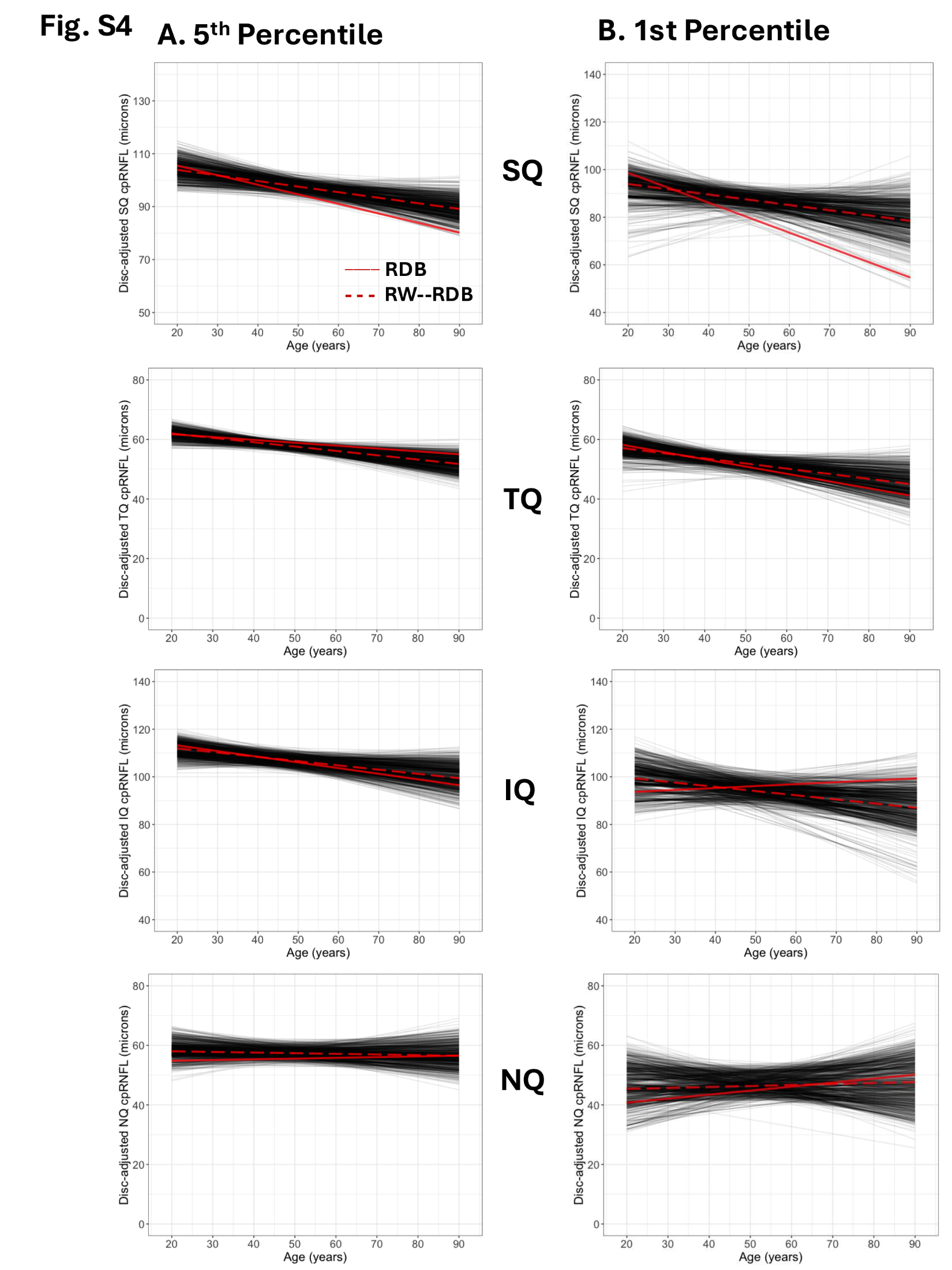
**

**Figure S4.** The thin black lines are the 5^th^ (A) and 1^st^ (B) pct QRLs for the 1000 samples of the disc-adjusted cpRNFL quadrant thickness. In all panels the solid red line represents the QRL for the 398 C-RDB, while the dashed red line represents the ORL for the RW-RDB.

**
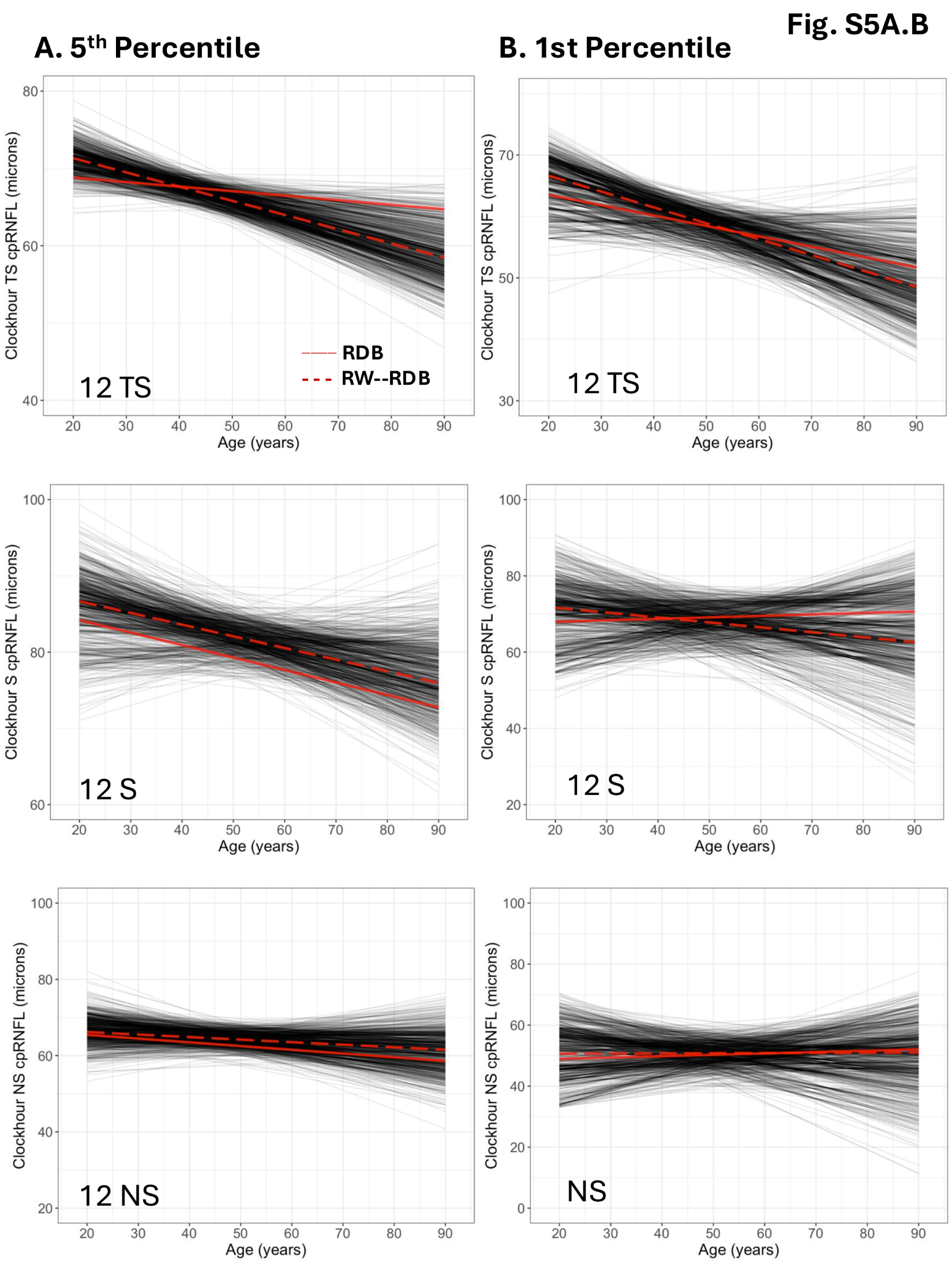
**

**
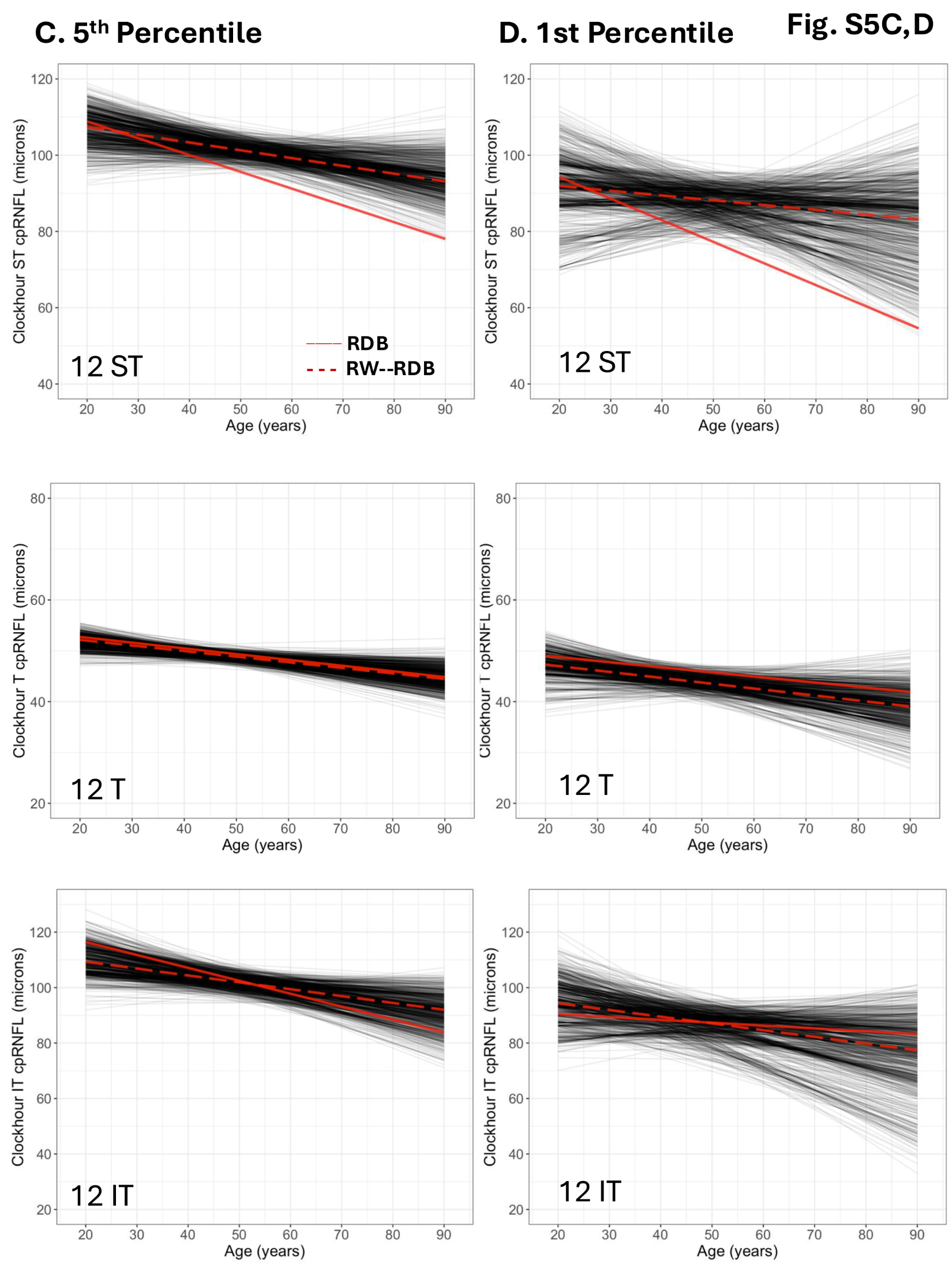
**

**
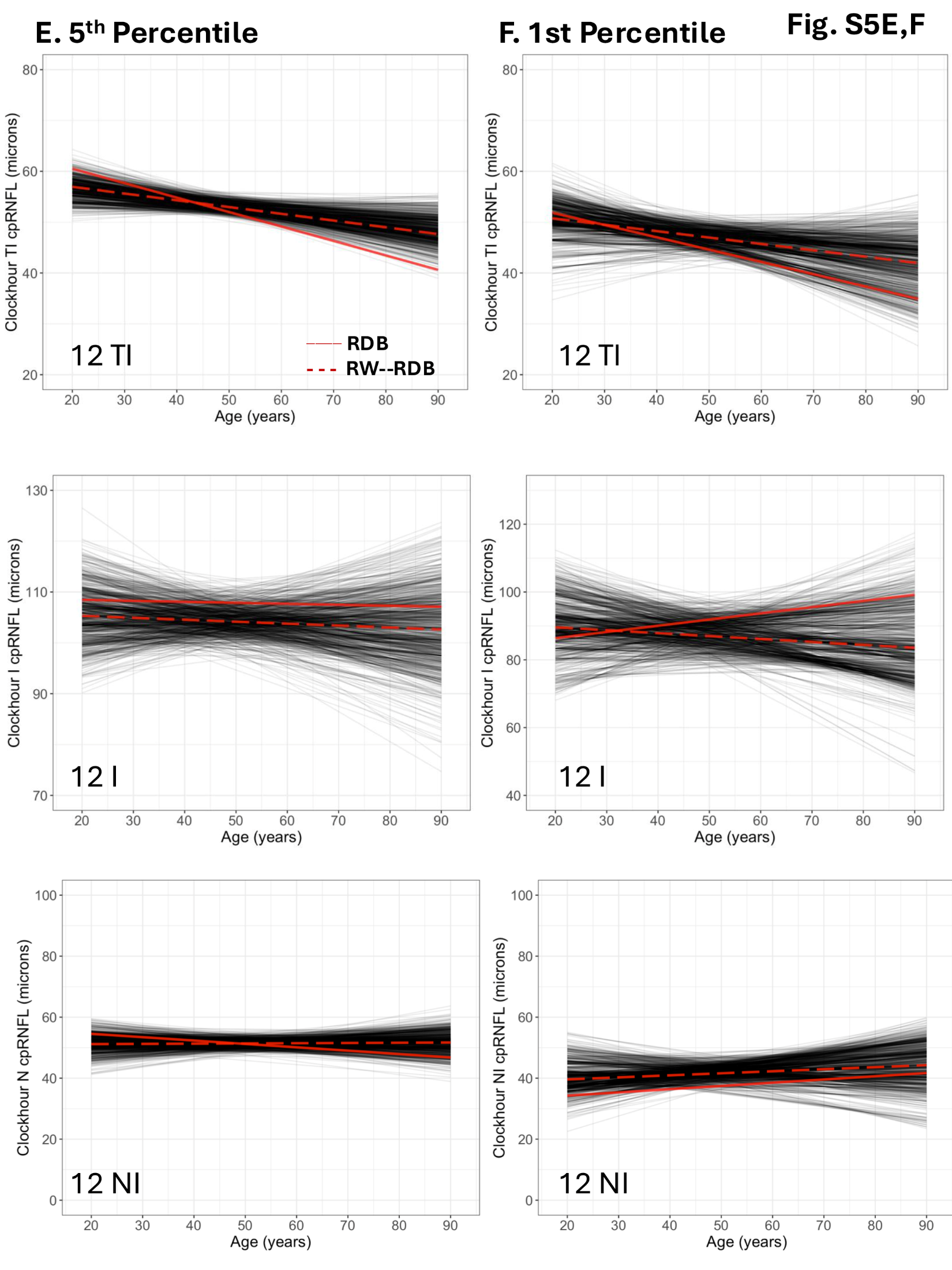
**

**
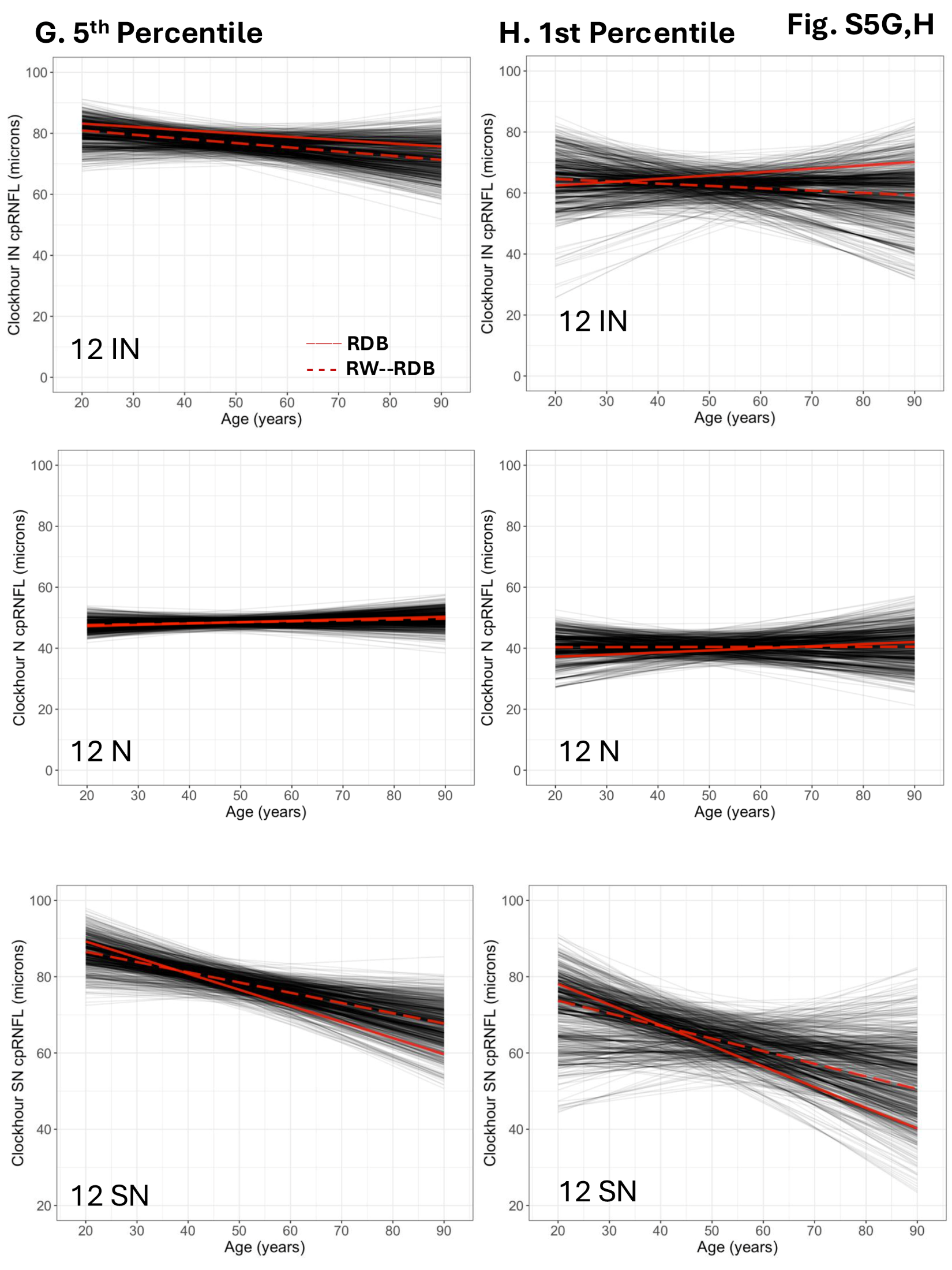
**

**Figure S5.** The thin black lines are the 5^th^ (A,C,E) and 1^st^ (B,D,F) pct QRLs for the 1000 samples of the disc-adjusted cpRNFL clock hour thickness. In all panels the solid red line represents the QRL for the 398 C-RDB, while the dashed red line represents the ORL for the RW-RDB.

**
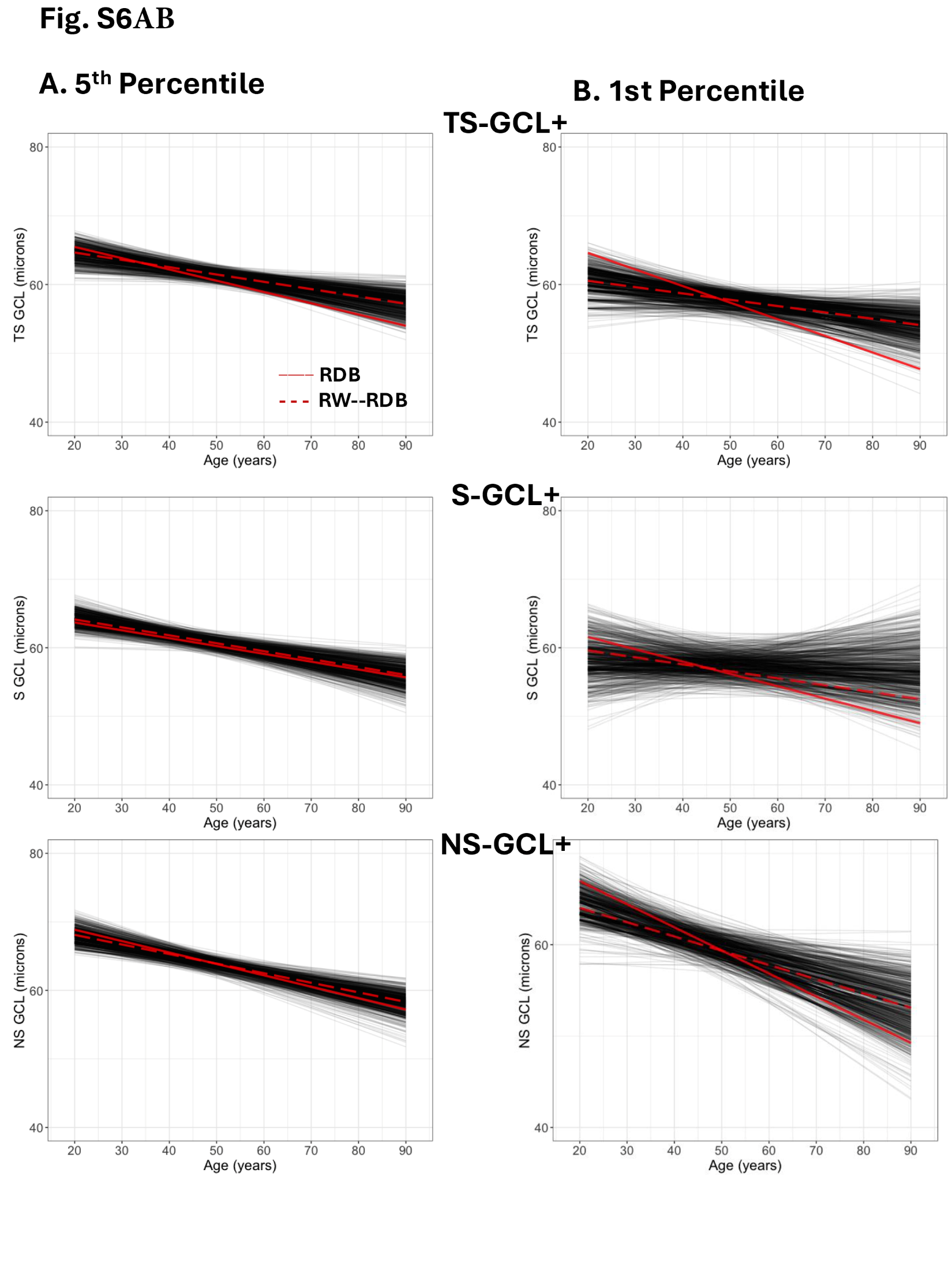
**

**
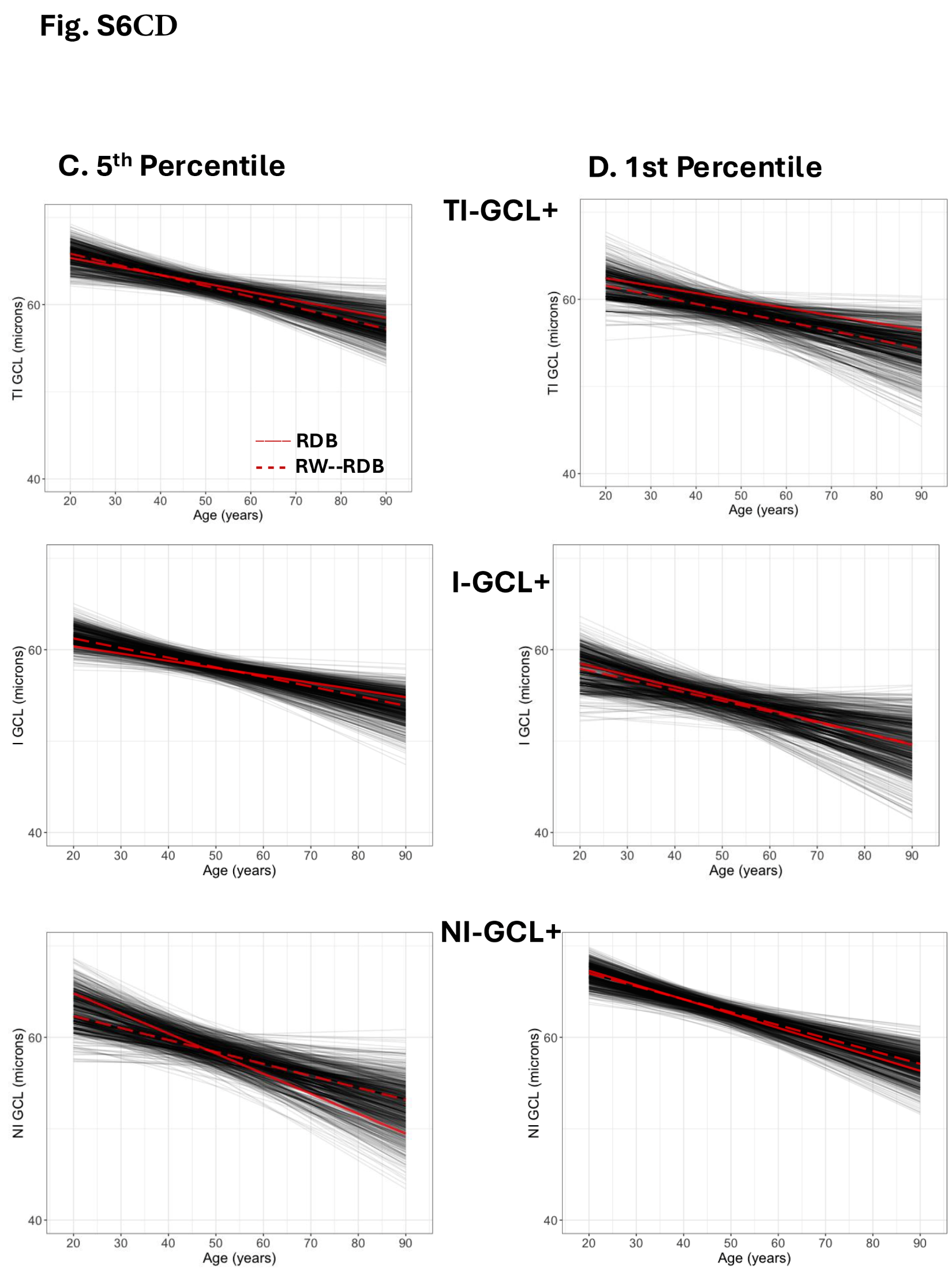
**

**Figure S6.** The thin black lines are the 5^th^ (A,C) and 1^st^ (B,D) pct QRLs for the 1000 samples of the GCL+ thickness of the 6 GCL sectors. In all panels the solid red line represents the QRL for the 398 C-RDB, while the dashed red line represents the ORL for the RW-RDB.
