# Supplementary material for: Larger Real-World OCT Reference Database Improves Accuracy of Glaucoma Flagging Using Summary Metrics
Source: Transl Vis Sci Technol. 2026 Mar 9;15(3):6. doi: 10.1167/tvst.15.3.6 (PMC12988682; doi:10.1167/tvst.15.3.6)
Supplement: Supplement 3 [file tvst-15-3-6_s003.docx]

| **Table S2. cpRNFL Clock Hour Thickness. Change in color-coding of 175 Healthy eyes** | | | | | | | | | | | |  |  |
| --- | --- | --- | --- | --- | --- | --- | --- | --- | --- | --- | --- | --- | --- |
| **398🡪4.8K** | | **T** | **TS** | **ST** | **S** | **SN** | **NS** | **N** | **NI** | **IN** | **I** | **IT** | **TI** |
| **G to Y** | | **0** | **1** | **4** | **3** | **3** | **2** | **0** | **1** | **0** | **0** | **1** | **0** |
| **Y to G** | | **0** | **1** | **0** | **0** | **0** | **0** | **0** | **4** | **2** | **2** | **2** | **1** |
| **Y to R** | | **0** | **0** | **2** | **1** | **0** | **0** | **0** | **0** | **0** | **0** | **0** | **1** |
| **R to Y** | | **1** | **0** | **0** | **0** | **0** | **0** | **0** | **0** | **0** | **1** | **0** | **0** |
| **Total** | | **1**  **0.6%** | **2**  **1.1%** | **6**  **3.4%** | **4**  **2.3%** | **3**  **1.7%** | **2**  **1.1%** | **0**  **0%** | **5**  **2.9%** | **2**  **1.1%** | **2**  **1.1%** | **2**  **1.1%** | **2**  **1.1%** |
| **Change in FPs** | **5%** | **0**  **0%** | **0**  **0%** | **4**  **-2.3%** | **3**  **-1.7%** | **3**  **-1.7%** | **2**  **-1.1%** | **0**  **0%** | **-3**  **1.7%** | **-2**  **1.1%** | **-2**  **1.1%** | **-1**  **0.6%** | **1**  **-0.6%** |
|  | **1%** | **-1**  **0.6%** | **0**  **0%** | **2**  **-1.1%** | **1**  **-0.6%** | **0**  **0%** | **0**  **0%** | **0**  **0%** | **0**  **0%** | **0**  **0%** | **-1**  **0.6%** | **0**  **0%** | **1**  **-0.6%** |
