# Supplementary material for: Larger Real-World OCT Reference Database Improves Accuracy of Glaucoma Flagging Using Summary Metrics
Source: Transl Vis Sci Technol. 2026 Mar 9;15(3):6. doi: 10.1167/tvst.15.3.6 (PMC12988682; doi:10.1167/tvst.15.3.6)
Supplement: Supplement 4 [file tvst-15-3-6_s004.docx]

| **Table s3. GCL+ Sector Thickness. Change in color-coding of 175 Healthy Eyes** | | | | | | | |
| --- | --- | --- | --- | --- | --- | --- | --- |
| **398🡪4.8K** | | **ST** | **S** | **NS** | **NI** | **I** | **IT** |
| **G to Y** | | **4** | **1** | **1** | **0** | **1** | **0** |
| **Y to G** | | **0** | **0** | **0** | **0** | **0** | **0** |
| **Y to R** | | **0** | **0** | **0** | **0** | **0** | **0** |
| **R to Y** | | **1** | **0** | **1** | **0** | **2** | **2** |
| **Total** | | **5**  **2.9%** | **1**  **0.6%** | **2**  **1.1%** | **0**  **0%** | **3**  **1.7%** | **2**  **1.1%** |
| **Change in FPs** | **5%** | **4**  **-2.3%** | **1**  **-0.6%** | **1**  **-0.6%** | **0**  **0%** | **1**  **-0.6%** | **0**  **0%** |
|  | **1%** | **-1**  **0.6%** | **0**  **0%** | **-1**  **0.6%** | **0**  **0%** | **-2**  **1.1%** | **-2**  **1.1%** |
