# Supplementary material for: Larger Real-World OCT Reference Database Improves Accuracy of Glaucoma Flagging Using Summary Metrics
Source: Transl Vis Sci Technol. 2026 Mar 9;15(3):6. doi: 10.1167/tvst.15.3.6 (PMC12988682; doi:10.1167/tvst.15.3.6)
Supplement: Supplement 5 [file tvst-15-3-6_s005.docx]

| **Table S4. cpRNFL Quadrant Thickness. Change in color-coding 183 ON-G eyes** | | | | | |
| --- | --- | --- | --- | --- | --- |
| **398🡪4.8K** | | **SQ** | **IQ** | **TQ** | **NQ** |
| **G to Y** | | **16** | **5** | **0** | **2** |
| **Y to G** | | **0** | **0** | **8** | **0** |
| **Y to R** | | **24** | **0** | **8** | **0** |
| **R to Y** | | **0** | **16** | **0** | **1** |
| **Total** | | **40**  **21.9%** | **21**  **11.5%** | **16**  **8.7%** | **3**  **1.6%** |
| **Change in TPs**  **(sensitivity)** | **5%** | **16**  **8.7%** | **5**  **2.7%** | **-8**  **-4.4%** | **2**  **1.1%** |
|  | **1%** | **24**  **13.1%** | **-16**  **-8.7%** | **8**  **4.4%** | **-1**  **-0.5%** |
