# Supplementary material for: Larger Real-World OCT Reference Database Improves Accuracy of Glaucoma Flagging Using Summary Metrics
Source: Transl Vis Sci Technol. 2026 Mar 9;15(3):6. doi: 10.1167/tvst.15.3.6 (PMC12988682; doi:10.1167/tvst.15.3.6)
Supplement: Supplement 6 [file tvst-15-3-6_s006.docx]

| **Table S5. cpRNFL Clock Hours. Change in color-coding of 183 ON-G eyes** | | | | | | | | | | | | | |
| --- | --- | --- | --- | --- | --- | --- | --- | --- | --- | --- | --- | --- | --- |
| **398🡪4.8K** | | **T** | **TS** | **ST** | **S** | **SN** | **NS** | **N** | **NI** | **IN** | **I** | **IT** | **TI** |
| **G to Y** | | **0** | **0** | **25** | **7** | **13** | **5** | **0** | **4** | **0** | **0** | **13** | **16** |
| **Y to G** | | **5** | **19** | **0** | **0** | **0** | **0** | **0** | **0** | **5** | **8** | **0** | **0** |
| **Y to R/GtoR** | | **0** | **0** | **46/1** | **0** | **7** | **0** | **0** | **5** | **0** | **0** | **0** | **10** |
| **R to Y** | | **5** | **4** | **0** | **0** | **0** | **0** | **1** | **0** | **13** | **16** | **2** | **0** |
| **Total** | | **5**  **2.7%** | **23**  **12.6%** | **72**  **39.3%** | **7**  **3.8%** | **20**  **10.9%** | **5**  **2.7%** | **1**  **0.5%** | **9**  **4.9%** | **18**  **9.8%** | **24**  **13.1%** | **15**  **8.2%** | **26**  **14.2%** |
| **Change in TPs**  **(sensitivity)** | **5%** | **-5**  **-2.7%** | **-19**  **-10.4%** | **25**  **13.7%** | **7**  **3.8%** | **13**  **7.1%** | **5**  **2.7%** | **0**  **0%** | **4**  **2.2%** | **-5**  **-2.7%** | **-8**  **-4.4%** | **13**  **7.1%** | **16**  **8.7%** |
|  | **1%** | **-5**  **-2.7%** | **-4**  **-2.2%** | **47**  **25.7%** | **0**  **0%** | **7**  **3.8%** | **0**  **0%** | **-1**  **-0.5%** | **5**  **2.7%** | **-13**  **7.1%** | **-16**  **8.7%** | **-2**  **-1.1%** | **10**  **5.5%** |
