# Supplementary material for: Larger Real-World OCT Reference Database Improves Accuracy of Glaucoma Flagging Using Summary Metrics
Source: Transl Vis Sci Technol. 2026 Mar 9;15(3):6. doi: 10.1167/tvst.15.3.6 (PMC12988682; doi:10.1167/tvst.15.3.6)
Supplement: Supplement 7 [file tvst-15-3-6_s007.docx]

| **Table S6. GCL+ Sector Thickness. Change in color-coding of 183 ON-G eyes** | | | | | | | |
| --- | --- | --- | --- | --- | --- | --- | --- |
| **398🡪4.8K** | | **ST** | **S** | **NS** | **NI** | **I** | **IT** |
| **G to Y** | | **19** | **5** | **3** | **2** | **0** | **0** |
| **Y to G** | | **0** | **0** | **0** | **0** | **4** | **6** |
| **Y to R** | | **18** | **9** | **2** | **7** | **0** | **0** |
| **R to Y** | | **0** | **0** | **0** | **0** | **1** | **22** |
| **Total** | | **37**  **20.2%** | **14**  **7.7%** | **5**  **2.7%** | **9**  **4.9%** | **5**  **2.7%** | **28**  **15.3%** |
| **Change in TPs**  **(sensitivity)** | **5%** | **19**  **10.4%** | **5**  **2.7%** | **3**  **1.6%** | **2**  **1.1%** | **-4**  **-2.2%** | **-6**  **-3.3%** |
|  | **1%** | **18**  **9.8%** | **9**  **4.9%** | **2**  **1.1%** | **7**  **3.8%** | **1**  **0.5%** | **22**  **12.0%** |
